# Three-Fingered RAVERs: Rapid Accumulation of Variations in Exposed Residues of Snake Venom Toxins

**DOI:** 10.3390/toxins5112172

**Published:** 2013-11-18

**Authors:** Kartik Sunagar, Timothy N. W. Jackson, Eivind A. B. Undheim, Syed. A. Ali, Agostinho Antunes, Bryan G. Fry

**Affiliations:** 1 CIMAR/CIIMAR, Centro Interdisciplinar de Investigação Marinha e Ambiental, Universidade do Porto, Rua dos Bragas, 177, Porto 4050-123, Portugal; E-Mails: anaturalist@gmail.com (K.S.); aantunes@ciimar.up.pt (A.A.); 2 Departamento de Biologia, Faculdade de Ciências, Universidade do Porto, Rua do Campo Alegre, Porto 4169-007, Portugal; 3 Venom Evolution Lab, School of Biological Sciences, The University of Queensland, St. Lucia, Queensland 4072, Australia; E-Mails: tnwjackson@gmail.com (T.N.W.J.); eivindandreas@gmail.com (E.A.B.U.); dr.syedabidali@gmail.com (S.A.A.); 4 Institute for Molecular Bioscience, The University of Queensland, St. Lucia, Queensland 4072, Australia; 5 HEJ Research Institute of Chemistry, International Center for Chemical and Biological Sciences (ICCBS), University of Karachi, Karachi-75270, Pakistan

**Keywords:** positive selection, venom evolution, three-finger toxins, RAVER, focal mutagenesis

## Abstract

Three-finger toxins (3FTx) represent one of the most abundantly secreted and potently toxic components of colubrid (Colubridae), elapid (Elapidae) and psammophid (Psammophiinae subfamily of the Lamprophidae) snake venom arsenal. Despite their conserved structural similarity, they perform a diversity of biological functions. Although they are theorised to undergo adaptive evolution, the underlying diversification mechanisms remain elusive. Here, we report the molecular evolution of different 3FTx functional forms and show that positively selected point mutations have driven the rapid evolution and diversification of 3FTx. These diversification events not only correlate with the evolution of advanced venom delivery systems (VDS) in Caenophidia, but in particular the explosive diversification of the clade subsequent to the evolution of a high pressure, hollow-fanged VDS in elapids, highlighting the significant role of these toxins in the evolution of advanced snakes. We show that Type I, II and III α-neurotoxins have evolved with extreme rapidity under the influence of positive selection. We also show that novel *Oxyuranus*/*Pseudonaja* Type II forms lacking the apotypic loop-2 stabilising cysteine doublet characteristic of Type II forms are not phylogenetically basal in relation to other Type IIs as previously thought, but are the result of secondary loss of these apotypic cysteines on at least three separate occasions. Not all 3FTxs have evolved rapidly: κ-neurotoxins, which form non-covalently associated heterodimers, have experienced a relatively weaker influence of diversifying selection; while cytotoxic 3FTx, with their functional sites, dispersed over 40% of the molecular surface, have been extremely constrained by negative selection. We show that the a previous theory of 3FTx molecular evolution (termed ASSET) is evolutionarily implausible and cannot account for the considerable variation observed in very short segments of 3FTx. Instead, we propose a theory of Rapid Accumulation of Variations in Exposed Residues (RAVER) to illustrate the significance of point mutations, guided by focal mutagenesis and positive selection in the evolution and diversification of 3FTx.

## 1. Introduction

Venoms are key evolutionary innovations in the Kingdom Animalia and are complex concoctions of biologically active proteins (from polypeptide globular enzymes to small peptides), salts, and organic molecules such as polyamines, amino acids and neurotransmitters [1]. Venom components originate via toxin recruitment events during which ordinary protein-encoding genes, typically those involved in key regulatory processes (such as hemostasis or neurotransmission) are duplicated, and the new copies are selectively expressed in the venom gland [1,2,3,4,5,6,7,8,9,10,11,12,13]. These novel paralogs can further duplicate and give rise to multigene families, following the “birth and death” mode of evolution, where the rapid evolution of these families results in extensive neofunctionalization of some copies, while the other non-functional forms are lost through degradation or get transformed into pseudogenes [14]. Research has shown that despite the extraordinary diversity of animal toxins, most belong to a limited number of enzymatic (e.g., phospholipases, serine proteases, metalloproteinases) and non-enzymatic (e.g., three-finger toxins, natriuretic peptides, Kunitz peptides, lectins) protein superfamilies which have been convergently recruited in various organisms to perform similar functions [1,3].

Three-finger toxins (3FTx) are one of the most abundantly secreted non-enzymatic components of elapid (Elapidae), colubrid (Colubridae) and psammophiide (Psammophiinae subfamily withing the Lamprophidae) snake venom. They are characterised by a broad diversity of functional forms (Table 1). In the past, 3FTx were considered to be exclusive to elapid snake venoms [6]. The discovery of α-colubritoxin, however, revealed this potent toxin type to be widespread in “non-front-fanged” (NFF) Caenophidia snake lineages [15]. Subsequent studies revealed the broad taxonomic distribution of this toxin type [16,17,18,19,20,21,22], which appears to have been recruited into the snake venom arsenal near the base of the snake tree [9]. 

3FTx are characterised by the presence of three β-loops that extend from the toxin’s small, hydrophobic core, giving them the “three-fingered” appearance and their name [23]. The plesiotypic (ancestral character state of a molecular scaffold) 3FTx form, such as that found in NFF advanced snakes, has ten cysteines in a distinctive pattern, reflective of its molecular origin from the recruitment of a LYNX/SLUR nicotinic receptor binding neuromodulation peptide [3,6,15,17]. All described 3FTx types from Henophidia, NFF and viperid (Viperidae) snakes contain these ten cysteines, and all of these forms that have been functionally investigated are α-neurotoxic, with a potency much greater against birds and/or reptiles than mammals [6,9,17,19,20,21,24]. This taxon specificity in action led to the plesiotypic α-neurotoxins being mistakenly referred to as “weak neurotoxins” (*c.f.* [25]). Similarly, this taxon-specific toxicity led to misinterpretation of prey-handling behaviour in an experiment investigating the role of venom in the feeding ecology of NFF snakes [26]. 

**Table 1:** Bioactivities of 3FTx types with characterised toxicities.

Functional Class	Mode of Action
Basal-type α-neurotoxins	Antagonists of α1 nicotinic acetylcholine receptors, with a 100-fold greater potency to avians/reptiles than mammals. Produces flaccid paralysis [21]
Type I α-neurotoxins	Antagonists of α1 nicotinic acetylcholine receptors. Produces flaccid paralysis [27]
Type II α-neurotoxins	Antagonists of α1 and α7 nicotinic acetylcholine receptors. Produces flaccid paralysis [27]
Type III α-neurotoxins	Antagonists of α1 nicotinic acetylcholine receptors. Produces flaccid paralysis [28]
κ-neurotoxins	Antagonists of α3β2 neuronal nicotinic acetylcholine receptor subtype. Produces flaccid paralysis [29]
Adrenergic/Muscarinic neurotoxins	Antagonists of a wide variety of adrenergic and muscarinic subtypes with extreme specificity for receptor subtypes [30,31,32,33,34,35,36,37,38,39,40,41]
Type B Muscarinic toxins	Antagonists of M2 muscarinic acetylcholine receptors [42]
ASIC channel blockers	Acts as a reversible gating modifier toxin by antagonistically binding to closed/inactivated ASIC1a-ASIC2a (ACCN2-ACCN1) channels in central neurons and ASIC1b-containing channels in nociceptors [43]
Calcium channel blockers	Antagonists of L-type calcium channels, thus inhibiting the transmission of the action potential [44]
Acetylcholinesterase inhibitors	Inhibit acetylcholinesterase through competitive binding [45]
Platelet inhibitors	Competitively bind to platelet GPIIb/IIIa receptor utilising the RGD functional motif, thus blocking platelet aggregation [46]
Cytotoxins	Cell-damaging activity mediated by hydrophobic-patch on molecular surface that interacts non-specifically with hydrophobic aspects of the cell phospholipid bilayer [47]
Synergistic	Alone are non-toxic but form complexes with α-neurotoxins to dramatically enhance neurotoxicity [48]

**Figure 1:**
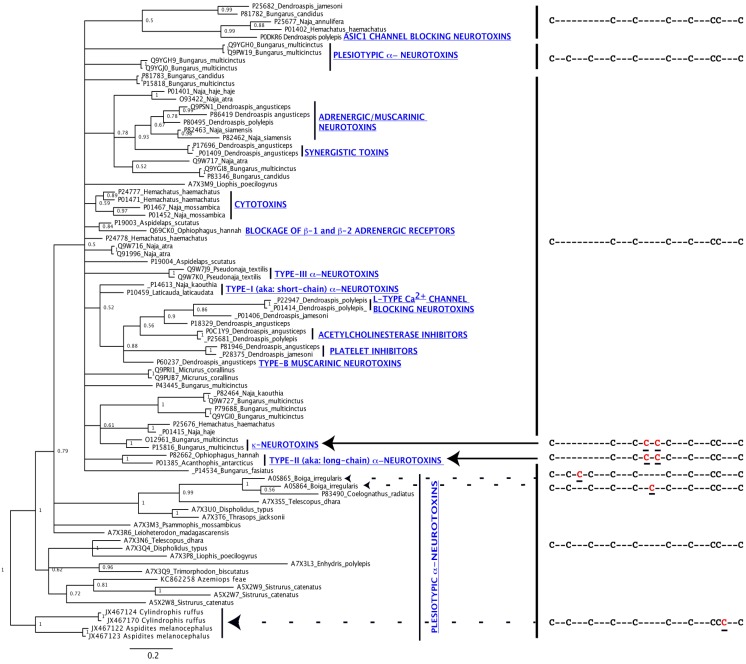
Bayesian molecular phylogeny of representative three-finger toxins. Uniprot [49] accession numbers are given for each. Cysteine framework variation is displayed, with ancestral cysteines in black and newly evolved cysteines in red.

Subsequent to elapid snakes evolving a high pressure, syringe-like delivery system including venom gland compressor musculature and hollow front fangs, apotypic (derived character state of a molecular scaffold) forms of 3FTx emerged, characterised by a loss of plesiotypic cysteines 2 and 3 (Figure 1), a change that probably resulted in the dramatic potentiation of α-neurotoxicity through the uncoupling of loop-1, with these apotypic forms becoming much more potent upon mammalian receptors than the more constrained plesiotypic forms [6]. This increased toxicity likely enhanced the role of these proteins in prey capture and resulted in a high level expression of α-neurotoxins (α-ntx) lacking the second and third plesiotypic cysteines (Type I (aka: short-chain) and Type II (aka: long-chain) α-ntx) in the venom glands of these snakes. The increased level of expression was accompanied by punctuated molecular evolution, resulting in the emergence of a myriad of structurally and functionally novel forms [6]. Structurally novel forms included α-ntx with newly evolved cysteines that stabilised the second loop (Type II α-ntx). Venoms of snakes from the *Oxyuranus* and *Pseudonaja* clade are unusual in containing significant amounts of 3FTx that display all the features of Type II α-ntx (including lacking the 2nd and 3rd plesiotypic cysteines) but lack the apotypic loop-2 stabilising cysteine pair characteristic of this type [50]. These toxins would thus be expected to be phylogenetically basal to the Type II α-ntx which contain the apotypic loop-2 stabilising cysteine pair. Another derived 3FTx structural variation is represented by the κ-neurotoxins (aka: κ-bungarotoxins), which form non-covalently associated heterodimers. A number of novel functions also emerged ([6]; Table 1; Figure 1), such as κ-ntx specifically targeting the neuronal nicotinic receptors. The most extreme neofunctionalisation is represented by the cytotoxins, which have deviated from the highly focused ion channel targeting of the α-ntx [51]. Instead, they exhibit a number of novel biological activities, including lysis of various types of cells (including erythrocytes and epithelial cells), enzyme inhibition (protein kinase C: [52]; Na+/K+ ATPase: [53]), depolarization and contraction of muscle cells and prevention of platelet aggregation. All structurally and functionally apotypic 3FTxs lack plesiotypic cysteines 2 and 3 (Figure 1), and are expressed in the venoms of elapid snakes in much higher levels than the α-ntxs that contain all ten plesiotypic cysteines.

Although it has been hypothesised and demonstrated through (now obsolete) selection assessments that snake venom three-finger toxins have evolved under the influence of positive Darwinian selection [6,54,55], the underlying mechanism of evolution driving the diversification of functional forms remains unclear. The molecular evolution of several 3FTx forms, such as κ-ntx, plesiotypic 3FTxs from Henophidia, NFF and elapid snakes and Type III α-neurotoxins from Australian elapids, remain unstudied to date. Molecular mechanisms underlying the evolution of the 3FTx gene in viperid snakes, which have independently evolved a sophisticated high pressure, hollow-fanged venom delivery system (VDS), also remain elusive. Moreover, interpretations regarding the evolution of certain 3FTx types, such as the suggestion that cytotoxins evolve under the influence of positive selection (with an ω value of 19.5) [55], have been questionable. Recently, the molecular evolution of 3FTx homologues in *Atractaspis sp.* was evaluated and the influence of positive Darwinian selection on genes encoding them was highlighted [56].

As the general organization of the 3FTx gene is highly conserved and ordered, Accelerated Segment Switch in Exons to alter Targeting (ASSET) has been postulated as the mechanism driving the molecular evolution of 3FTx [16,57]. This theory suggests that during the evolution of the 3FTx gene, segments in exonic regions have been exchanged with distinctly different ones and the resultant “switching” of segments has generated the observed sequence variation and the functional diversity of 3FTx. The authors further speculated that point mutations alone could not account for the diversity of 3FTx functional forms and that they could only be helpful in fine tuning receptor binding capabilities, which originally arise through ASSET [16,57]. However, this study attempted to classify regions in 3FTx based on simplistic “degree of identity” comparisons. Crucially, such analysis does not take into account the fact that proteins adopt regionally differential rates of evolution [58,59,60]. It has been previously demonstrated in other toxin types that structurally important residues are constrained by negative selection, while regions responsible for biological function and/or those forming the molecular surface accumulate variations under an arms race scenario [58,59,61]. This not only facilitates functional diversification, but also increases the number of active residues on the surface of venom components that can non-specifically interact with novel receptors in prey and induce a plethora of pharmacological effects.

In the present study, we test the following hypotheses: (i) that 3FTx with specific sites of action are involved in a coevolutionary arms race with receptors of prey animals and thus are evolving under the influence of positive selection; (ii) that cytotoxic 3FTx, which interact non-specifically with cell membranes, do not experience an arms race and evolve under the constraints of negative selection pressure; (iii) that, in venoms in which 3FTx are a major component (Elapidae, Colubridae and Psammophiinae), 3FTx are rapidly evolving under positive selection (with the exception of cytotoxic 3FTx in the venom of elapid snakes); (iv) that in venoms in which 3FTx are a minor component (Viperidae), 3FTx evolve under a neutral selection regime and do not experience positive selection; and (v) the evolution of the advanced venom delivery system in elapid snakes led to an increase in diversifying pressure on 3FTx, resulting in the diversity of functional forms present in the venoms of snakes from this family. In order to test these hypotheses and provide further insight into the evolution and diversification of this toxin superfamily, we reconstructed the complex molecular evolutionary history of 3FTx. In particular, this study examined: the relative rate of evolution of the plesiotypic α-ntx (i) before and after the evolution of the advanced venom delivery apparatus in Caenophidia (advanced snakes), in order to test whether the evolution of a sophisticated VDS influenced the regime of 3FTx evolution; (ii) before and after the evolution of the sophisticated, high pressure and hollow-fanged venom delivery system in elapids and viperids to test whether the refining of VDS influenced selection pressures on 3FTx genes; (iii) the relative rate of 3FTx evolution subsequent to the loss of two plesiotypic cysteines and resultant potentiation of α-neurotoxicity; (iv) the relative rate of evolution subsequent to the apotyposis (or derivation) of non-covalently associated dimeric forms, in order to find out whether these new structural constraints affected natural selection pressures on this gene; and finally; (v) the relative rate of evolution subsequent to the apotyposis of cytotoxins was examined to understand whether the novel activity recruited by cytotoxins affected their rate of evolution.

## 2. Results

Bayesian and maximum-likelihood molecular phylogenetic analyses retrieved phylogenetic trees with similar topologies (Figure 1; Supplementary Figure 1), with different toxin sequences forming distinct phylogenetic clades as shown previously [3,6,17]. The Bayesian tree (convergence diagnostics: Average standard deviation of split frequencies: 0.009; PSRF = 1.00) was built using amino acid sequences, since some 3FTxs are only known from their amino acid sequences, while the maximum-likelihood tree (1,000 bootstrap replicates; GTR + I + G) was built using all the nucleotide sequences analysed in this study (*n* = 457). The overall topology of these phylogenetic trees was largely in concordance with the previously published 3FTx phylogeny [3,6,17].

**Figure 2:**
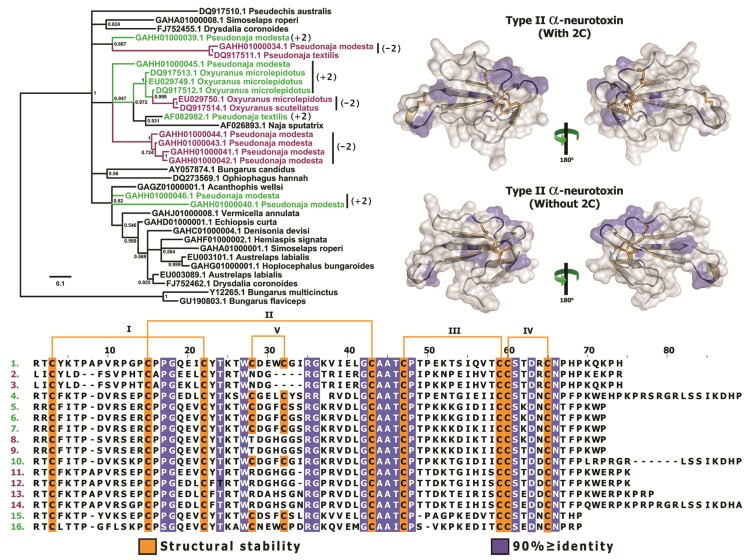
Bayesian molecular phylogeny and structural and functional evolution of +2C/−2C Type II (long-chain) α-neurotoxins. *Pseudonaja*/*Oxyuranus* −2C and +2C sequences are coloured purple and green, respectively. Sequences presented (uniprot): (1) R4FIT5 *Pseudonaja modesta*, (2) R4FIU6 *Pseudonaja modesta*, (3) A8HDK6 *Pseudonaja textilis*, (4) R4FK68 *Pseudonaja modesta*, (5) A8HDK8 *Oxyuranus microlepidotus*, (6) A7X4Q3 *Oxyuranus microlepidotus*, (7) A8HDK7 *Oxyuranus microlepidotus*, (8) A7X4R0 *Oxyuranus microlepidotus*, (9) A8HDK9 *Oxyuranus scutellatus*, (10) Q9W7J5 *Pseudonaja textilis*, (11) R4FIT0 *Pseudonaja modesta*, (12) R4G7K3 *Pseudonaja modesta*, (13) R4G321 *Pseudonaja modesta*, (14) R4G2J4 *Pseudonaja modesta*, (15) R4G319 *Pseudonaja modesta* and (16) R4FK75 *Pseudonaja modesta*.

A particularly notable finding was that the novel Type II α-ntx sequences from *Oxyuranus* and *Pseudonaja*, which lack the Type II α-ntx characteristic cysteine doublet (−2C) between plesiotypic cysteines 5 and 6, were not phylogenetically basal to the other Type II, nor were they monophyletic, but rather were nested within the regular Type II forms (+2C) (Figure 2). This suggests that the apotypic cysteine doublet characteristic of Type II α-ntx was secondarily lost on at least three occasions. 

Our analysis of the venom gland transcriptome of the unique viperid species *Azemiops feae* recovered a 3FTx transcript that was found in the same clade as the representative sequences from the pit-viper *Sistrurus catenatus* (Figure 1; Supplementary Figure 1). This is the first non-*Sistrurus* 3FTx to be recovered from a viperid snake. Based on an unresolved neighbour joining tree, viperid 3FTx were previously reported as polyphyletic, with some sequences nested within elapid 3FTx clade, and others in the “non-front-fanged” advanced snake 3FTx clade [24]. However, both Bayesian and maximum-likelihood phylogenetic analyses in this study place all viperid sequences in a monophyletic clade outside elapid 3FTx clade with strong node support (Figure 1; Supplementary Figure 1). This is consistent with the viperid snakes having diverged from the remaining advanced snakes nearly 54 million years ago [62]. viperid 3FTx homologs retrieved to date indicate that apotypic structural and functional forms, such as Type I, II and III α-ntxs, κ-ntxs and cytotoxins, are the result of apotyposis in elapid snakes after this split (Figure 1; Supplementary Figure 1).

The one-ratio model (ORM), the simplest of the lineage-specific models, computed a wide range of ω (dN/dS) values for various types of 3FTx (Supplementary Tables 1–10). This highly conservative model can only detect positive selection when the ω ratio, averaged over all sites along the lineages in a phylogenetic tree, is significantly greater than one. Despite this, the ω value for most three-finger toxins was significantly greater than 1, indicating the strong influence of positive selection in shaping the evolution of 3FTx: plesiotypic α-ntx from viperids, NFF and elapids: 1.79, 1.29 and 1.30, respectively; Type I, Type II and Type III α-ntx: 1.92, 2.01 and 2.59, respectively; and κ-ntx: 1.64. *Oxyuranus/Pseudonaja* Type II α-ntx with (+2C) and without the cys doublet (−2C) were significantly different, with ω values of 0.97 and 2.69, respectively. In contrast to all other 3FTx types, ORM estimated an ω value of 0.32 for cytotoxins, indicating an unprecedented lack of variation in this unique three-finger toxin.

The segregation of seromucous mixed maxillary glands into discrete protein and mucus glands at the base of the advanced snake tree and the subsequent evolution of a high pressure, hollow-fanged venom delivery apparatus independently in *Atractaspis*/*Homoroselaps*, elapids and viperids were major evolutionary advances in snake-feeding ecology [7,17]. In order to assess the effect of apomorphic (derived state of a morphological character) venom delivery systems on the evolution of 3FTx, we employed the three-ratio model (3RM) and the branch-site test A (BST) on the plesiotypic α-ntx from the advanced snakes (NFF, viperids and elapids). While 3RM—which assesses selection pressure only across lineages—estimated ω of 1.13, 1.37 and 2.21 for the plesiotypic α-ntx from NFF, elapids and viperids, respectively; BST—which assess selection pressures across the sites and along the lineages in a tree—estimated ω of 2.16 (22% sites), 4.09 (23% sites) and 6.54 (31% sites), respectively (Table 2). NFF and elapid comparisons were insignificant (*p* > 0.05) for 3RM, while all other comparisons were significant (significant at 0.001 even after Bonferroni corrections). 3RM and BST indicated a greater influence of positive selection on the plesiotypic α-ntxs in elapid and viperid snakes, which have independently evolved sophisticated high pressure and hollow-fanged venom delivery systems.

**Table 2:** Lineage-specific analyses of plesiotypic Caenophidia three-finger toxins (3FTxs).

Model	ω ^a^	Likelihood (ι)	Prop. of Sites with ω > 1 ^b^	Significance ^c^
**Plesiotypic α-neurotoxins from ‘non-front-fanged’ advanced snakes**				
Three-ratio model	1.13	−5982.232579	-	*P* > 0.05 ^NS^
Branch-site model A	2.16	−5767.710454	22.1%	* *P* << 0.001
Clade model C	2.20	−5670.149246	49.9%	* *P* << 0.001
**Plesiotypic α-neurotoxins from Elapidae**				
Three-ratio model	1.37	−5984.727017	-	*P* > 0.05^NS^
Branch-site model A	4.09	−5773.846707	22.6%	* *P* << 0.001
Clade model C	4.48	−5670.149246	49.9%	* *P* << 0.001
**Plesiotypic α-neurotoxins from Viperidae**				
Three-ratio model	2.21	−5981.804559	-	* *P* << 0.001
Branch-site model A	6.54	−5762.853194	31.1%	* *P* << 0.001
Clade model C	6.23	−5670.149246	49.9%	* *P* << 0.001

**Legend: ^a^:** mean dN/dS; **^b^:** Proportion of sites with ω > 1; **^c^:** Significance of the model in comparison with the null model; * **:** Bonferroni corrected values; **NS:** Not significant (at 0.05).

We also employed these tests to assess selection pressures along the lineages of the three types of elapid-specific apotypic α-ntx that lack plesiotypic cysteines 2 and 3: Type I, II and III α-ntxs (Table 3). The three-ratio model (3RM) estimated ω of 1.89, 1.94 and 2.39 for Type I, Type II and Type III α-ntxs, respectively. BST estimated ω of 4.39 (10% sites), 4.09 (18% sites) and 4.77 (24% sites), respectively for the three types. All these comparisons were significant at 0.001 even after Bonferroni corrections.

**Table 3:** Lineage-specificanalyses of α-neurotoxins.

Model	ω ^a^	Likelihood (ι)	Prop. of Sites with ω > 1 ^b^	Significance ^c^
Type I α-neurotoxins				
Three-ratio model	1.89	−12,395.465265	-	* *P* << 0.001
Branch-site model A	4.39	−11,868.198045	9.8%	* *P* << 0.001
Clade model C	3.36	−11,602.547050	44.4%	* *P* << 0.001
Type II α-neurotoxins				
Three-ratio model	1.94	−12,395.367372	-	* *P* << 0.001
Branch-site model A	4.09	−11,751.045621	18.3%	* *P* << 0.001
Clade model C	4.14	−11,602.547050	44.4%	* *P* << 0.001
Type III α-neurotoxins				
Three-ratio model	2.39	−12,394.644636	-	* *P* << 0.001
Branch-site model A	4.77	−11,812.912890	23.7	* *P* << 0.001
Clade model C	3.26	−11,602.547050	44.4%	* *P* << 0.001

**Legend: ^a^:** mean dN/dS; **^b^:** Proportion of sites with ω > 1; **^c^:** Significance of the model in comparison with the null model; *: Bonferroni corrected values; **NS:** Not significant (at 0.05).

**Figure 3:**
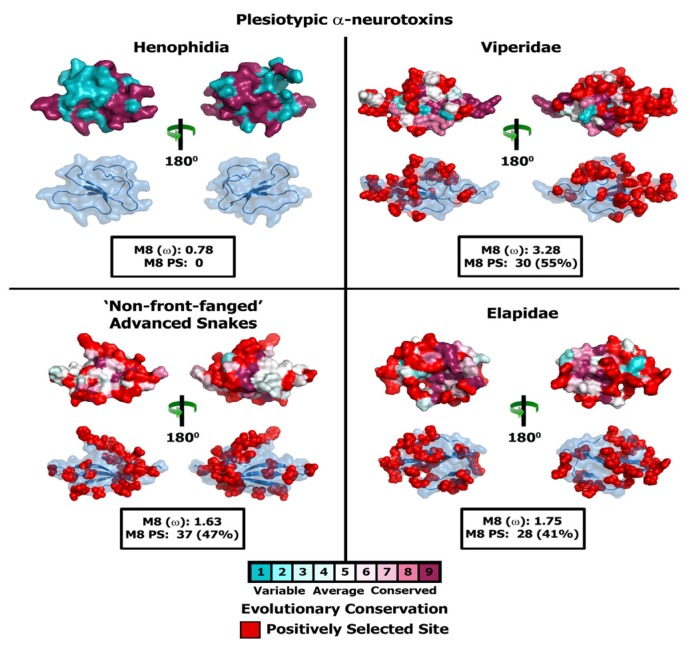
Molecular evolution of plesiotypic 3FTxs. Three-dimensional homology models of various three-finger toxins (top: surface-fill; bottom: wireframe), depicting the locations of positively selected sites are presented. Site-model 8 computed omega and the total number of positively selected sites detected by its Bayes Empirical Bayes (BEB) approach (*PP* ≥ 0.95) are also indicated.

**Figure 4:**
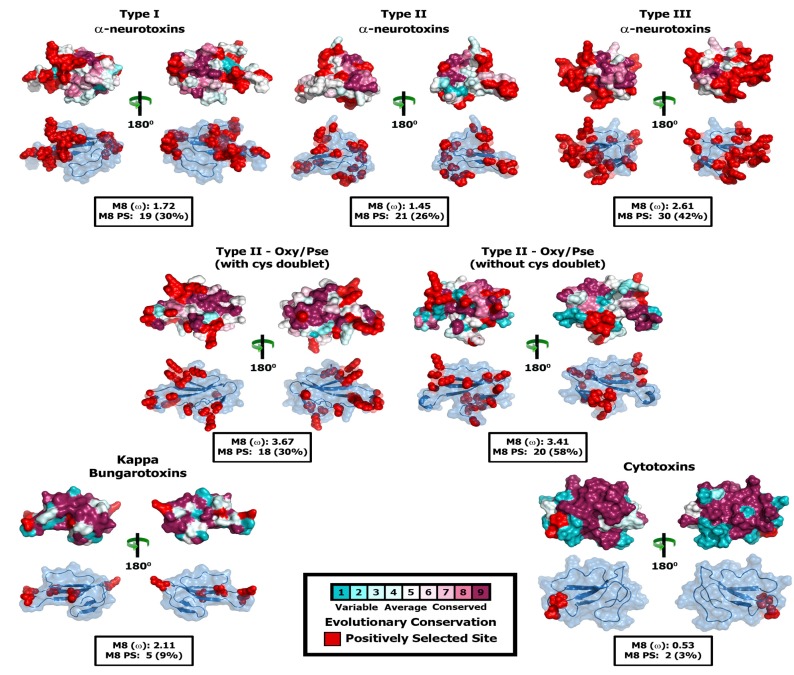
Molecular evolution of derived 3FTx. Three-dimensional homology models of various three-finger toxins (top: surface-fill; bottom: wireframe), depicting the locations of positively selected sites are presented. Site-model 8 computed omega and the total number of positively selected sites detected by its Bayes Empirical Bayes (BEB) approach (*PP* ≥ 0.95) are also indicated.

Since the aforementioned lineage-specific models often fail to detect episodic diversifying selection that only affects certain sites in proteins, we also employed site-specific models. Similar to ORM, model 8 estimated ω that ranged widely (Table 4; Figure 3 and Figure 4; Supplementary Tables 1–10). The computed ω and the number of positively selected sites detected by model 8 suggest that most three-finger toxins have evolved under the strong influence of positive selection; viperids: 3.28 (30 positively selected (PS) sites; 55% of sites)); plesiotypic α-ntx from NFF: 1.63 (37 PS; 47% of sites); plesiotypic α-ntx from elapid snakes: 1.75 (28 PS; 41% of sites); Type I α-ntx: 1.72 (19 PS; 30.0% of sites); Type II α-ntx: 1.45 (21 PS; 26% of sites); *Oxyuranus/Pseudonaja* Type II α-ntx with cys doublet (+2C): 3.67 (18 PS; 33% of sites); *Oxyuranus/Pseudonaja* Type II α-ntx without cys doublet (−2C): 3.41 (20 PS; 58% of sites); Type III α-ntx 2.61 (30 PS; 42% of sites); κ-bungarotoxins: 2.11 (5 PS; 9.3% of sites) (Table 4; Figure 3 and Figure 4; Supplementary Tables 1–10). FUBAR and MEME identified several episodically diversifying sites in various forms of 3FTxs, highlighting the rapid evolution of these toxins (Table 4). In contrast, M8 estimated ω of 0.53 (2 PS and 3% of sites) for cytotoxic 3FTx, suggesting that they have evolved under a regime of strong negative selection. Compared to the α-ntxs, κ-bungarotoxins have experienced a weaker influence of positive diversifying selection, as reflected by the very small number of positively selected sites detected in this lineage by various analyses (5PS and 9% of sites; Table 4).

**Table 4:** Dynamic molecularevolution of three-finger toxins (3FTx).

	FUBAR ^a^	MEME ^b^	BSR ^c^	PAML ^d^	
		#sites	#branches	M8	M2a
**Plesiotypic α-neurotoxins**					
**Henophidia 3FTx**	ω > **1^e^**: 0 ω < **1^f^**: 0	0	0	0	0
				ω: 0.78	ω: 0.78
**Non-front-fanged advanced snake 3FTx**	ω > **1^e^**: 18 ω < **1^f^**: 7	18	9	37	26
				(27 + 10)	(18 + 8)
				ω: 1.63	ω: 1.71
**Elapidae 3FTx**	ω > **1^e^**: 13 ω < **1^f^**: 3	6	8	28	24
				(22 + 6)	(15 + 9)
				ω: 1.75	ω: 1.79
**Viperidae 3FTx**	ω > **1^e^**: 7 ω < **1^f^**: 1	0	6	30	24
				(18 + 12)	(13 + 11)
				ω: 3.28	ω: 3.28
**Apotypic or Derived α-neurotoxins**					
**Type I α-neurotoxin**	ω > **1^e^**: 22 ω < **1^f^**: 6	13	9	19	13
				(13 + 6)	(11 + 2)
				ω: 1.72	ω: 1.61
**Type II α-neurotoxin**	ω > **1^e^**: 21 ω < **1^f^**: 11	30	24	21	19
				(19 + 2)	(17 + 2)
				ω: 1.45	ω: 1.43
**Type III α-neurotoxin**	ω > **1^e^**: 29 ω < **1^f^**: 4	24	13	30	27
				(26 + 4)	(26 + 1)
				ω: 2.61	ω: 2.59
***Oxyuranus*/*Pseudonaja* derived Type II α-neurotoxins**					
**With-Cysteine- Doublet**	ω > **1^e^**: 11 ω < **1^f^**: 2	6	7	18	17
				(11 + 7)	(10 + 7)
				ω: 3.67	ω: 3.57
**Without-Cysteine-Doublet**	ω > **1^e^**: 5 ω < **1^f^**: 1	1	2	20	14
				(9 + 11)	(2 + 12)
				ω: 3.41	ω: 3.41
**Cytotoxins**	ω > **1^e^**: 1 ω < **1^f^**: 2	7	0	2	2
				(2 + 0)	(1 + 1)
				ω: 0.53	ω: 0.57
**Kappa neurotoxins**	ω > **1^e^**: 6 ω < **1^f^**: 0	3	2	5	5
				(3 + 2)	(3 + 2)
				ω: 2.11	ω: 2.11

**Legend:** **^a^:** Fast Unconstrained Bayesian AppRoximation; **^b^:** Sites detected as experiencing episodic diversifying selection (0.05 significance) by the Mixed Effects Model Evolution (MEME); **^c^:** Number of branches detected by the branch-site REL (Random effects likelihood) test as episodically diversifying (not comparable to positively selected sites); **^d^:** Positively selected sites detected by the Bayes Empirical Bayes approach implemented in M8 and M2a. Sites detected at 0.99 and 0.95 significance are indicated in the parenthesis (total number of positively selected sites is also indicated along with the estimated ω); **^e^:** number of sites under pervasive diversifying selection at the posterior probability ≥0.9 (FUBAR); **^f^:** Number of sites under pervasive purifying selection at the posterior probability ≥0.9 (FUBAR); ω: mean dN/dS.

Since lineages may have different proportions of sites under the influence of selection, the direct comparison of ω values computed by the aforementioned analyses could be misleading. Hence, we employed the clade model c approach to simultaneously compute ω values for different 3FTx clades. Clade-model analyses computed ω of 2.20, 4.48 and 6.23 for the plesiotypic α-ntxs from NFF, elapid and viperid snakes, respectively (Table 2). This highlights a greater influence of positive selection on 3FTx from elapid and viperid snake lineages (significant at 0.001 in comparison with site model M1a, even after Bonferroni correction), relative to NFF and Henophidia 3FTxs. Within elapid snake specific 3FTx lineages, clade analyses estimated ω of 3.36, 4.14 and 3.26 for Type I, Type II and Type III α-ntxs, respectively, indicating a greater influence of positive Darwinian selection on Type II α-ntx compared to the rest (Table 3).

To provide further support to the positively detected sites by the nucleotide-level selection analyses (M8), we employed a complementary protein-level approach implemented in TreeSAAP. With this combined approach we were able to identify 18/19 positively selected (PS) sites in Type I, all 21 PS sites in Type II, 13/30 PS sites in Type III α-ntxs; 21/30 sites in viperid 3FTx; 11/37 PS sites in NFF and 2/2 PS sites in cytotoxic 3FTx (Supplementary Table 11: highlighted in bold). However, this approach failed to identify positively selected sites in κ-bungarotoxins and elapid plesiotypic 3FTx lineages and +2C and −2C type II α-ntx lineages, suggesting that mutations in these toxin types may not be as radical as the ones in the aforementioned types.

**Figure 5:**
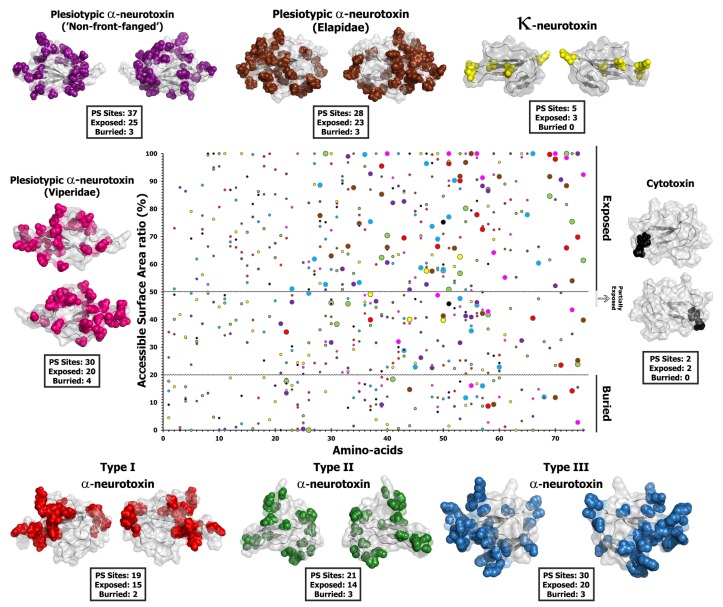
Surface accessibility of three-finger toxins. A plot of amino acid positions (x-axis) against accessible surface area (ASA) ratio (y-axis) indicating the locations of amino acids (exposed or buried) in the crystal structure of various three-finger toxins is presented. Positively selected residues are presented as large dots, while the remaining sites are presented as small dots in the plot. Residues with an ASA ratio greater than 50% are considered to be exposed (ASA of 40%–50% are likely to have exposed side chains), while those with a ratio lesser than 20% are considered to be buried to the surrounding medium. Three-dimensional structures of various 3FTx types, depicting the locations of positively selected (PS) sites along with model 8 omega values and the number of exposed and buried positively selected sites are also presented (for details see Table 5).

**Figure 6:**
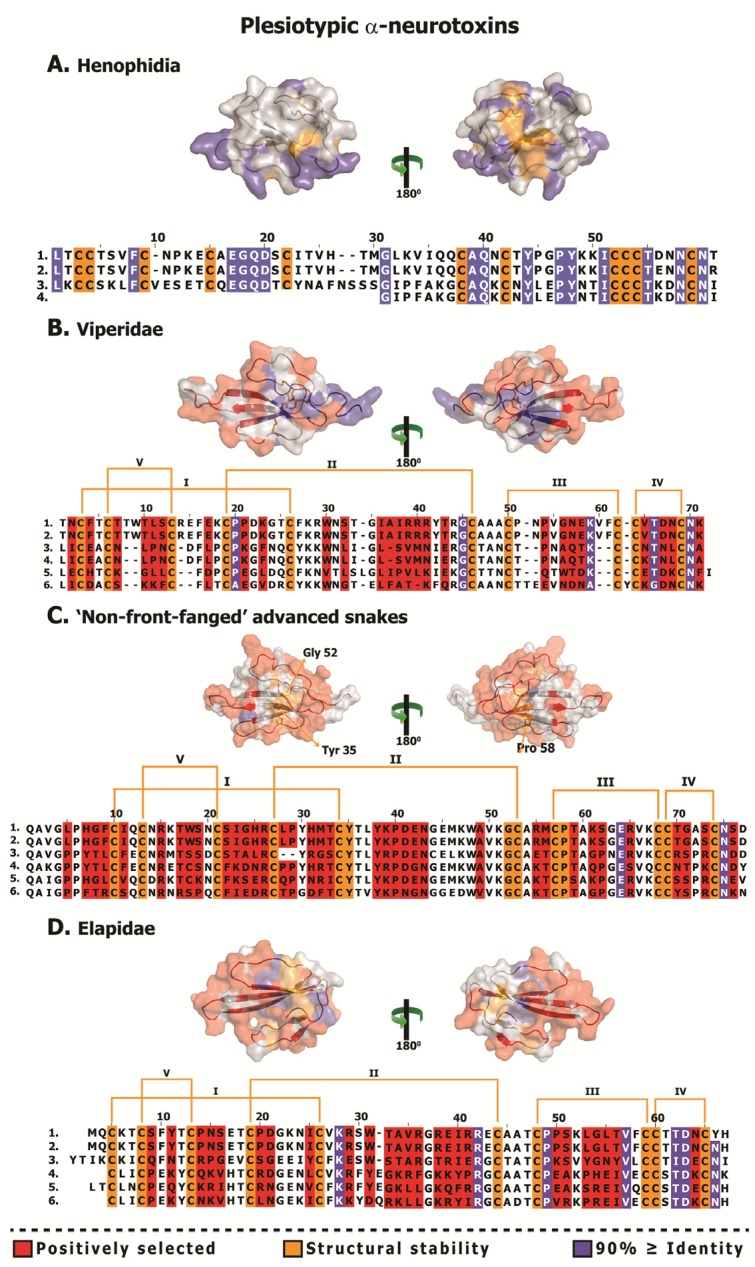
Structural and functional evolution of plesiotypic 3FTxs. Sequence alignment and homology models depicting structurally and functionally important residues and hypermutable sites of various three-finger toxins are shown. Extremely well-conserved residues implicated in structural/functional roles, and hypermutable sites are shaded. Sequences presented (uniprot): **A.** (1) M9T1L2 *Aspidites melanocephalus*, (2) M9T271 *Aspidites melanocephalus*, (3) M9SZR1 *Cylindrophis ruffus* and (4) M9SZV9 *Cylindrophis ruffus*; **B.** (1) A5X2W8 *Sistrurus catenatus edwardsii*, (2) B4Y146 *Sistrurus catenatus edwardsii*, (3) A5X2W7 *Sistrurus catenatus edwardsii*, (4) B4Y143 *Sistrurus catenatus edwardsii*, (5) B4Y144 *Sistrurus catenatus edwardsii* and (6) M9T2J4 *Azemiops feae*; **C.** (1) Q06ZW0 *Boiga dendrophila*, (2) A0S864 *Boiga irregularis*, (3) A0S865 *Boiga irregularis*, 4) A7X3V0 *Telescopus dhara* and (5) A7X3S0 *Trimorphodon biscutatus*; **D.** (1) Q6IZ95 *Bungarus candidus*, (2) Q9PW19 *Bungarus multicinctus*, (3) Q9YGH9 *Bungarus multicinctus*, (4) Q8AY51 *Bungarus candidus*, (5) Q2VBN2 *Ophiophagus hannah* and 6) Q9YGI2 *Naja atra*.

**Figure 7:**
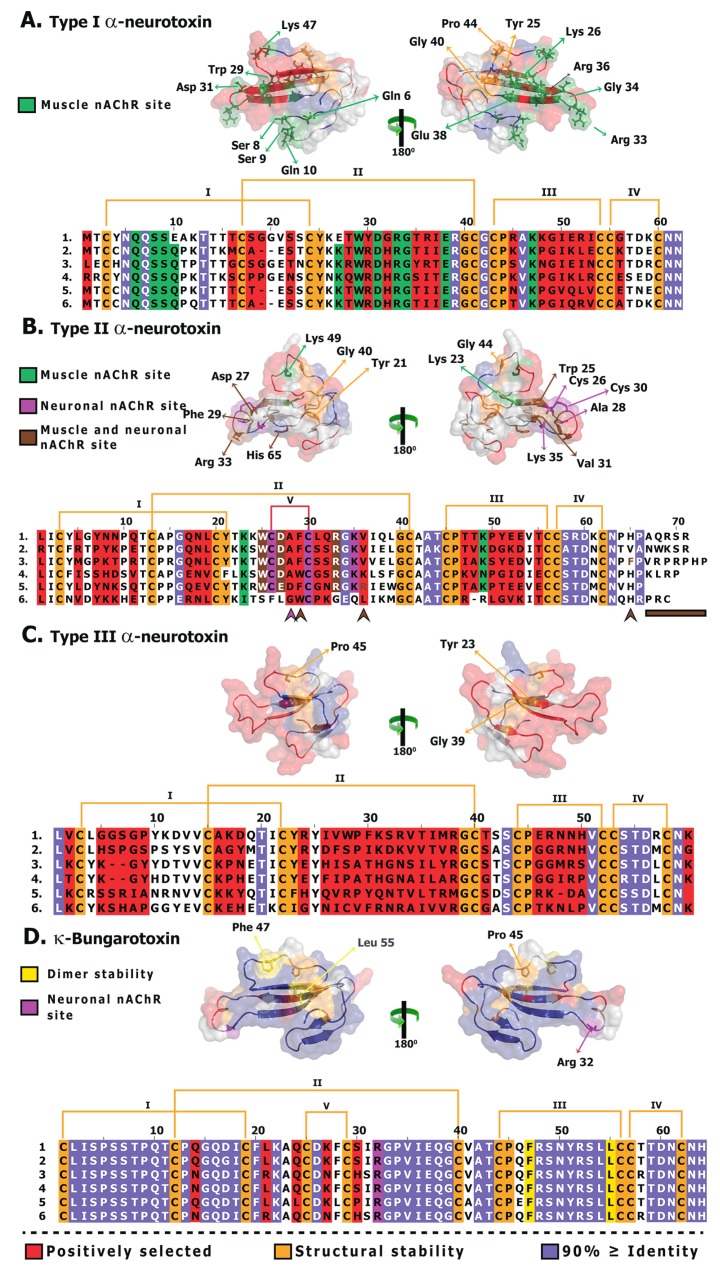
Structural and functional evolution of derived α-neurotoxins and κ-neurotoxins. Sequence alignment and homology models depicting structurally and functionally important residues and hypermutable sites of various three-finger toxins are shown. Extremely well-conserved residues implicated in structural/functional roles, and hypermutable sites are shaded. Sites that are shown to be structurally/functionally important but exhibit less than 70% identity are indicated by arrow heads in the alignment. Sequences presented (uniprot): (**A**). (1) B5G6F6 *Oxyuranus microlepidotus*, (2) H8PG58 *Suta nigriceps*, (3) P60770 *Naja atra*, (4) P10455 *Laticauda colubrina*, (5) B2BRS2 *Austrelaps labialis* and (6) F8J2H2 *Drysdalia coronoides*; (**B**). (1) F8J2E0 *Drysdalia coronoides*, (2) Q8UW29 *Hydrophis hardwickii*, (3) P01384 *Notechis scutatus*, (4) P82662 *Ophiophagus hannah*, (5) R4G2L3 *Suta fasciata* and (6) R4G2E5 *Brachyurophis roperi*; **C.** (1) R4G2S0 *Cacophis squamulosus*, (2) R4G7H6 *Furina ornata*, (3) R4G332 *Pseudonaja modesta*, (4) Q9W7K2 *Pseudonaja textilis*, (5) R4G7F3 *Brachyurophis roperi* and (6) R4G7M0 *Vermicella annulata*; **D.** (1) Q8AY56 *Bungarus candidus*, (2) Q8AY55 *Bungarus candidus*, (3) P15817 *Bungarus multicinctus*, (4) P01398 *Bungarus multicinctus*, (5) O12962 *Bungarus multicinctus* and (6) Q9W729 *Bungarus multicinctus*.

**Figure 8:**
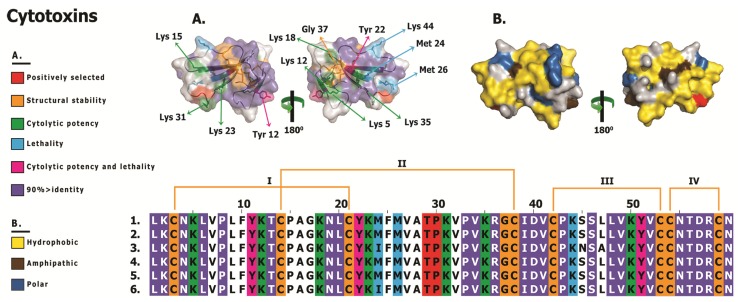
Structural and functional evolution of cytotoxins. Sequence alignment and homology models depicting **A.** the locations of putative functional residues, and **B.** hydrophobic regions of cytotoxins are presented. Extremely well-conserved residues implicated in structural/functional roles, and hypermutable sites are shaded. Sequences presented (uniprot): (1) P60301 *Naja atra*, (2) P60303 *Naja kaouthia*, (3) Q9DGH9 *Naja kaouthia*, (4) P60301 *Naja sputatrix*, (5) Q02454 *Naja sputatrix* and (6) O93471 *Naja sputatrix*.

Evolutionary fingerprint analyses clearly depict several residues in most 3FTx as evolving under the influence of positive selection, while conversely the majority of sites in cytotoxic 3FTx remain constrained under negative selection (Supplementary Figure 2). Branch-site tests assume that a phylogenetic tree can be divided into foreground (branches experiencing positive selection) and background (branches evolving under neutral or negative selection) lineages. It has been demonstrated that these tests can suffer from high rates of false-positives and false-negatives when such modeling assumptions are violated [63]. Hence, we employed the branch-site REL (BSR) test, which does not make such assumptions and outperforms branch-site tests. This test identified a number of branches in the phylogenetic trees of various 3FTx types as episodically diversifying: plesiotypic α-ntx from NFF, elapid and viperid snakes: 9, 8 and 6 branches, respectively; Type I, Type II and Type III α-ntx 9, 24 and 13 branches, respectively; +2C and −2C *Oxyuranus/Pseudonaja* Type II α-ntx: 7 and 2 branches, respectively (Supplementary Figures 3–5). This test detected two and three branches, respectively in κ-ntxs and cytotoxins, indicating that even these toxin types have experienced the influence of episodic bursts of selection, probably during their early stages of evolution (Supplementary Figure 6). Computation of accessible surface area ratio indicated that a majority of positively selected sites detected by M8 in 3FTx were exposed (completely or partially) to the surrounding medium (Figure 5; Supplementary Tables 11). The likelihood of a hypermutable site to be exposed to the surrounding medium was 2 to 3.5 times greater, compared to the likelihood of it being buried (Table 5). Mapping of positively selected sites onto the alignment of various 3FTx genes revealed that nearly all the hypermutable sites are located outside the structurally and functionally important regions, suggesting that 3FTx genes are subjected to a very sophisticated focal mutagenesis (Figure 2, Figure 6, Figure 7 and Figure 8; Supplementary Table 12).

**Table 5:** Relative frequency of positively selected sites.

Toxin Type	Total Residues	Exposed Total *	Exposed PS	Exposed PS %	Buried Total *	Buried PS	Buried PS %	Ratio Exposed % PS:Buried % PS
Plesiotypic NFF	74	40	25	62.5	17	3	17.6	**3.5**
Plesiotyic Viperidae	72	40	22	55%	20	4	20	**2.8**
Plesiotypic Elapidae	65	39	23	59	14	3	21.4	**2.8**
Type I α-ntx	62	38	15	39.5	10	2	20	**2**
Type II α-ntx	74	38	14	36.8	20	3	15	**2.5**
Type III α-ntx	57	34	20	58.8	14	3	21.4	**2.7**
κ-ntx	64	35	3	8.5	17	0	-	**N.A.**
Cytotoxic 3FTx	60	34	2	5.9	15	0	-	**N.A.**

PS = positively selected; N.A. = not applicable; NFF: ‘non-front-fanged’ advanced snakes; Ntx: Neurotoxin; * Some residues were ambiguous as being exposed or buried and thus were not assigned to either category.

## 3. Discussion

### 3.1. Evolution of an Advanced Venom Delivery Apparatus and Subsequent Evolution of a High Pressure, Hollow-Fanged Venom Delivery System May Have Facilitated Rapid Evolution of Three-Finger Toxins.

The early evolution of a venom system has been shown to have played a significant role in the diversification of Toxicofera reptiles [9,17,64,65,66,67] and the morphology and development of the venom delivery system has been shown to be intimately linked to feeding ecology/prey-handling behaviour [68,69,70,71,72,73,74,75,76,77]. This relationship between venom system morphology and feeding ecology has been discussed in detail elsewhere [7,78,79,80,81]. While some Henophidia do not use constriction for prey capture (e.g., *Cylindrophis*) and have mandibular and maxillary venom glands that are predominantly protein secreting, others (such as Boidae and Pythonidae) are not dependent on envenoming, and instead use an apomorphic strategy (constriction) for prey subjugation [9]. In contrast to Henophidia snakes, elapid and viperid snakes possess sophisticated venom delivery apparatus that facilitate efficient injection of venom into the prey. Various selection analyses employed in this study indicated that these contrasting prey capture strategies and the evolution of the advanced venom-delivery apparatus in Caenophidia have significantly influenced the evolution of the plesiotypic α-ntxs (Figure 3 and Figure 6; Table 2 and Table 4). Not surprisingly, plesiotypic α-ntxs in NFF (*ω* = 1.63 and 7 Positively Selected (PS) sites), viperid (*ω* = 3.28 and 30 PS) and elapid (*ω* = 1.75 and 28 PS) accumulated tremendous amount of variations, while their Henophidia homologs (*ω* = 0.77 and 0 PS) lacked variation completely (Table 2 and Table 4; Figure 3 and Figure 6). This is consistent with 3FTx variation being unnecessary in powerfully constricting snakes, which are not known to use venom for capturing prey [9]. Interestingly, the Henophidiae genera *Aspidites* (Pythonidae) and *Cylindrophis* (Cylindrophiidae) are separated by ~92 million years of evolution, almost as long as their common ancestor has been separated from the advanced snakes (approximate date of divergence 103 mya: [62]). Extreme conservation of 3FTx in the largely mucus-secreting maxillary and mandibular glands of these basally divergent snakes [9], despite their 90+ million years of independent evolution, is noteworthy. Due to the paucity of 3FTx sequences (primarily a result of their extremely low levels of venom gland specific transcription), the precise role of these proteins in the venom glands of Henophidia and the reason for the lack of variation in their coding sequences remains unclear.

The significant amount of variation observed in the plesiotypic α-ntxs from the venoms of NFF is consistent with high expression levels in the Colubridae and Psammophiinae [17,18]. 3FTx from several NFF snakes have been demonstrated to be potently toxic, and in some cases exhibit prey-specific toxicity [6,20,21,82]. However, various selection analyses presented in this study indicate a greater accumulation of variations in the plesiotypic α-ntxs from viperids and elapids, relative to the NFF snake α-ntx homologs: Table 2 and Table 4; Figure 3 and Figure 6; Supplementary Tables 1–3; Supplementary Figures 2 and 3). The sophisticated venom delivery apparatus of elapids facilitates efficient injection of the toxin cocktail into prey animals. Thus, the evolution of the high pressure, hollow-fanged venom delivery apparatus appears to have had a considerable influence on the rapid diversification of elapid and viperid 3FTxs in comparison with NFF snakes. The polyphyletic NFF snakes have been demonstrated to employ a characteristically different venom arsenal from that of elapid snakes. The venom in most elapid snakes is rich in three-finger toxins, while only the venoms of gracile snakes in Colubridae or Psammophiinae have been shown to contain large amounts of this toxin type [6,17,18]. Hence, the differences in overall venom composition could have also influenced the differential rate of 3FTx evolution in these lineages. It has already been demonstrated that some venom components that are secreted in large amounts by NFF snakes experience a greater influence of positive selection relative to those of their elapid snake counterparts [58]. Since most viperid snakes are known to employ a haemorrhagic, haemolytic toxin cocktail, the neurotoxic 3FTx may only play an ancillary role in envenoming by these snakes. Evidently, viperid snake venoms are dominated by hydrolytic enzymes (such as snake venom metalloproteinases and serine proteases), while 3FTx expression appears to occur only in insignificant amounts. Hence, the presence of considerable coding sequence variation in the viperid 3FTx gene is puzzling. Further sequencing and functional assessment of 3FTx from this lineage is warranted to precisely understand the participation of these proteins in envenoming.

### 3.2. The Deletion of Plesiotypic Cysteine Resides (2 and 3) May Have Triggered an Explosive Diversification

Comparative assessments revealed that although all three forms of apotypic α-ntxs that lack the 2nd and 3rd plesiotypic cysteines have evolved particularly rapidly, Type II forms have experienced a greater influence of positive selection than Type I or Type III α-ntxs (Table 3 and Table 4; Figure 4 and Figure 7; Supplementary Tables 4–6; Supplementary Figure 4A,B,C). Unlike Type I α-ntx that can only antagonise the muscle nicotinic acetylcholine receptors (α_1_-nAChR) (the plesiotypic functional activity), Type II α-ntxs can strongly bind to neuronal α_7_-nACh receptors, as well. This expanded target potential makes them extremely useful for prey paralysis and probably explains the greater influence of positive selection on this type of 3FTx. Type III α-ntx has been demonstrated to exhibit activities similar to that of Type I α-ntxs, but at a lower potency, in experiments conducted on mice [28]. Prey-specific toxicity has been demonstrated in a variety of α-ntxs [20,21,82]. Hence, Type III α-ntx in the venoms of Australian elapids could be less potent against mammals, but extremely potent against the organisms on which they specialise, such as lizards [83,84,85,86,87]. The majority of Type III α-ntxs have been recovered from venom gland transcriptomes of small Australian elapid snakes that feed exclusively on reptiles. The possibility that this toxin type may exhibit taxon-specific toxicity should be investigated further, as it may account for the tremendous influence of positive selection on this toxin type.

### 3.3. The Dramatic Rate of Evolution of Type II α-Neurotoxins May Have Resulted in the Loss of Characteristic Cysteine Doublets on Multiple Occasions in Oxyuranus/Pseudonaja

*Oxyuranus* and *Pseudonaja,* two closely related genera of Australian elapid snakes with unusually potent venom, are known to express the regular Type II α-ntx (+2C) and an additional type that lacks the two apotypic cysteine residues involved in stabilising loop 2 (−2C). It was previously suggested that the absence of the cysteine pair (−2C) was the phylogenetically basal form and that the Type II α-ntx form with the cysteine pairs (+2) represented the more derived form [88]. However, our molecular phylogenetic analyses revealed that the −2C originate from within the larger Type II α-ntx clade and thus represent secondary losses of the apotypic loop-2 stabilising cysteine pair (Figure 1 and Figure 2; Supplementary Figure 1). Type II α-ntxs have experienced a strong influence of positive selection pressure and some of these in *Oxyuranus* and *Pseudonaja* species have accumulated mutations even in structurally important regions that are typically constrained by negative-selection. As a result, on at least three occasions, they have lost the two apotypic cysteine residues that are characteristic of Type II α-ntxs (Table 3 and Table 4; Figure 2 and Figure 4; Supplementary Figures 4B and 5). Taxon-specific variations in potency of the +2C *versus* −2C have not been investigated but such studies would be illuminating.

### 3.4. Not All Three-Finger Toxin Clades Evolve via Pervasive Positive Darwinian Selection

Evidence provided by various analyses [Codeml site, branch, branch-site and clade-specific models: M8 and M2a, three-ratio model, branch-site test A, clade model c; HyPhy: MEME, FUBAR, Branch-site REL; TreeSAAP: protein-level selection analyses and evolutionary fingerprint analyses (Table 2, Table 3, and Table 4; Figure 2, Figure 3, Figure 4, Figure 5, Figure 6, and Figure 7; Supplementary Tables 1–9; Supplementary Figures 2–6)], clearly demonstrates the substantial influence of positive diversifying selection on the evolution of various three-finger toxin types. Conspicuously, while most 3FTx lineages evolved rapidly, cytotoxins (*ω* = 0.53; 2 PS-sites) remain constrained by negative selection (Table 4; Figure 4 and Figure 8; Supplementary Figures 2 and 6; Supplementary Table 10). The precise molecular mechanism by which cytotoxic 3FTx exert their cell-damaging effects remains unclear, but it has been proposed that a highly conserved hydrophobic patch on the cytotoxin’s molecular surface interacts with hydrophobic regions of the cell’s phospholipid bilayer [89,90]. Hydrophobic residues that facilitate such non-specific interactions are spread over the three loops of the cytotoxins (Figure 8: Panel B) and cover nearly 40% of the molecular surface. Additionally, cytotoxins are known to exhibit a diversity of biological functions and the residues implicated in these activities are distributed all along the length of the toxin sequence (Figure 8; Supplementary Table 10). In contrast to α-ntx, which bind to specific receptor sites, cytotoxins bind in a much more non-specific manner and thus may escape the classic predator–prey chemical arms race that influences the diversification of other toxin types. Thus, a lack of variation within the cytotoxins is reflective of the tremendous negative selection pressure on their structurally and functionally important residues, which further prevents the loss of activity and/or potency. In contrast, α-neurotoxins and an array of the ion channels they target, likely exert reciprocal selection pressure upon each other which results in the diversification of the 3FTx gene, in a classic “Red Queen” coevolutionary arms race scenario.

Conversely, κ-ntxs (*ω* = 2.11; 5 PS-sites) have accumulated fewer variations than the α-ntxs (Table 4; Figure 4 and Figure 7: Panel D; Supplementary Figures 2 and 6), despite also targeting a narrowly defined ion-channel type [91]. The lack of variations in the κ-ntxs could be attributed to their unique ability amongst 3FTx to form non-covalent dimers [91]. Thus, most residues in these toxins evolve under the influence of negative selection, preventing the accumulation of mutations that might affect the three-dimensional structure of the toxin, and thus the ability to non-covalently form dimers. It is noteworthy that κ-ntxs are characterised by two stabilising cysteines in loop 2, which maintain a more rigid three dimensional structure and are homologous to the same apotypic disulfide-bond in loop II of the Type II α-ntxs. Besides the van der Waal’s bond between Phe47 and Leu55 (Figure 7: Panel D), as many as six main chain and three side chain hydrogen bonds are known to stabilise this toxin [92]. The necessity to form these bonds exerts a strong structural constraint against the accumulation of variation in κ-ntxs. Thus variation in κ-ntx is largely limited to five positively selected sites, most of which are found in loop II. Loop III and the region between C9–C10, along with the functionally/structurally important residues evolve under the constraints of negative selection (Figure 7: Panel D).

α-ntxs and κ-ntxs have been theorised to originate from a common ancestral 3FTx subtype gene [6,93]. They are characterised by the highly conserved signal peptide and 3’ non-coding regions and share an apotypic loop-2 stabilising cysteine pair [93,94,95,96]. Moreover, comparison of the structural motifs in type II α-ntxs and the κ-ntxs, particularly the residues around the shared ten cysteines (eight plesiotypic plus the apotypic loop-2 stabilising pair), indicates how closely related the two genes are (Figure 7: Panels B and D). Hence, it is apparent that they have originated from a common ancestral 3FTx subtype gene and the differential rate of evolution has resulted in the observed functional and structural differences between the two homologs. In contrast to α-ntx, κ-bungarotoxin sequences are very well conserved, as a result of the aforementioned structural constraints (Figure 7: Panel D).

### 3.5. Rapid Accumulation of Variations in Exposed Residues (RAVER) and Focal Mutagenesis in the Evolution of Snake Venom Three-Finger Toxins

Considering the highly conserved organization of the exonic regions of 3FTx, Doley *et al*. 2008 [16] developed a hypothesis of “Accelerated Segment Switch in Exons to alter Targeting” (ASSET), to explain the molecular mechanism of 3FTx evolution. They postulated that during the evolution of the 3FTx gene, segments in exonic regions have been exchanged with distinctly different ones and the resultant “switching” of segments has generated the coding sequence variation and the diversity of 3FTx. The molecular mechanism of segment “switching” was attributed to a complex scenario involving genetic recombination, splicing variation, and independent recruitment events. We argue that these suggested mechanisms are unlikely to have played a major role in the evolution of three-finger toxins. Recombination cannot account for the tremendous variations observed in extremely short segments of 3FTx; all the observed hypermutations were concentrated in 1–6 adjacent amino acid sites (Figure 6, Figure 7, and Figure 8). Furthermore, recombination would not discriminate between functionally/structurally important and unimportant residues. All 3FTx have highly conserved residues (with eight of the original ten plesiotypic cysteines conserved in all forms), involved in maintaining functional and/or structural stability, and none of the hypermutable sites coincide with these (Figure 6, Figure 7, and Figure 8; Supplementary Table 12). In contrast, evidence supporting the hypothesis that all 3FTx genes originated from the same ancestral gene is strong (Figure 1; Supplementary Figure 1; [15]). Hence, the suggestion of multiple independent recruitment of 3FTx as means to explain the observed variations is implausible. Though alternate splicing or the errors associated with this process can introduce variation in toxin length, like recombination, they cannot account for the considerable variations observed in very short segments of 3FTx. In addition, insertions and/or deletions in shared molecular scaffolds are more plausible explanations for the variations in sequence length.

The role of point mutations in shaping the diversity of 3FTx gene was underestimated by the aforementioned authors [57]. They speculated that point mutations alone could not account for the diversity of 3FTx functional forms and could have only been helpful in fine-tuning receptor binding capabilities, which arise through ASSET [16,57]. A prominent role for point mutations in the evolution of 3FTx gene was discounted based on the following reasoning: (i) point mutations would require numerous generations to account for the observed variations; (ii) all intermediates would have to be selected for via positive selection; and (iii) point mutations would have to occur independently in unrelated lineages to produce the same structurally/functionally important conserved motif. 

We argue that these reasons for rejection are not valid. Predatory venom-encoding genes have been demonstrated to evolve under the significant influence of positive Darwinian selection, coupled with focal mutagenesis [6,59,97]. They are likely involved in a coevolutionary arms race, where evolving venom resistance in prey and the evolution of novel venom components exert reciprocal selective pressures on one another [98,99]. The recruitment of the 3FTx gene to reptilian venom systems was previously thought to have occurred within Caenophidia snakes between the divergence of the pareatid snakes approximately 65 million years ago (MYA) and that of the viperid snakes approximately 54 MYA [62]. We have recently recovered 3FTx transcripts from the venom glands of Henophidia snakes, demonstrating that this recruitment event in fact occurred within the ancient Afrophidia snake clade *circa* 104 MYA [9]. 3FTx genes explosively diversified subsequent to the evolution of the advanced venom delivery apparatus in elapids, 40 MYA (Table 2, Table 3, and Table 4; Figure 3, Figure 4, Figure 5, Figure 6, Figure 7, and Figure 8; Supplementary Figures 2–8). Therefore, an appropriately large number of generations have passed for point mutations, under the influence of positive Darwinian selection to shape the diversity of 3FTxs and account for the tremendous variation observed.

The aforementioned study [16] also failed to take into account the essence of focal mutagenesis, in which point mutations in certain regions of the toxins, such as the molecular surface, may confer an adaptive advantage. In order to conserve catalytic functions, most mutations in proteins concentrate in structurally and/or functionally unimportant regions. Since mutations occur randomly, they are equally likely to occur even within important regions of proteins. However, mutations in structurally or functionally important regions could disrupt the normal functioning of the protein and hence they are filtered out of the population by natural selection. Synthesis and secretion of venom proteins is an energetically expensive process [100,101,102] and hence, natural selection will only permit the rapid accumulation of mutations in certain regions of the toxin, such as the molecular surface, where accumulation of variation could confer an adaptive advantage. Evidently, venom proteins have been demonstrated to evolve via mutation of the surface chemistry, which introduces novel residues (and side-chains) on the molecular surface that could *non-specifically* interact with the novel targets in prey animals and cause a myriad of pharmacological effects [58,59,103]. In certain organisms like vampire bats, focal mutagenesis in venom proteins can be advantageous as it is likely to delay/prevent the development of immunological resistance in the prey by introducing extremely variable protein surface chemistry in the vampire bat population [61]. Not surprisingly, in vampire bats it has been previously demonstrated that prolonged feeding can result in mounting of immunological resistance in the prey [104], further evidence of the predator–prey (or parasite–host) arms race in which toxins are involved. Mapping of mutations onto three-dimensional homology models and computation of accessible surface area ratios for various three-finger toxins, suggested that a greater proportion of mutations have accumulated on the molecular surface and in the loops of these toxins (Figure 2, Figure 3, Figure 4, Figure 5, Figure 6, Figure 7, and Figure 8; Supplementary Tables 11). We found that the likelihood of hypermutable sites in three-finger toxins occurring on the molecular surface was greater than the likelihood of the site being buried (Table 5).

Hence, we propose a new theory called: “Rapid Accumulation of Variations in Exposed residues” (RAVER) to explain the molecular evolution of the 3FTx gene. We theorise that most predatory venom proteins experience RAVER or focal mutagenesis, which preserves the structural and functional integrity of the toxin, while permitting the rapid accumulation of variations on the molecular surface through positive selection. Thus, while variations accumulate on the molecular surface of the toxin under a coevolutionary arms race scenario, rare mutations in structural and functional residues of rapidly evolving types generate novel molecular scaffolds that get selected only if they considerably improve the potency of the toxin in comparison with the plesiotypic form. Otherwise, such mutations may get eliminated from the population by negative selection. The frequency at which such “rare” mutations accumulate within a functional type likely depends on the strength of positive Darwinian selection. Indeed, rapid accumulation of point mutations under the influence of positive selection in Type II α-ntx gene has led to the origination of −2C Type II α-ntxs on multiple occasions in *Oxyuranus* and *Pseudonaja,* two closely related genera of Australian elapid snakes with unusually potent venom (Figure 2).

### 3.6. Structure–Function Relationship in Three-Finger Toxins

Mapping positively selected sites onto alignments of various three-finger toxins revealed that a large number of residues implicated in structural/functional roles were extremely well conserved (80% ≥ identity; Figure 7 and Figure 8; Supplementary Table 12). However, very few functionally/structurally important residues (A28, F29, R36 and F65) in Type II α-ntxs have been poorly conserved. This is not surprising given the rapid evolution of Type II α-ntxs, which has even resulted in the replacement of two cysteine residues that are usually invariant, and led to the formation of new toxin type (−2C Type II α-ntxs) in *Oxyurnaus* and *Pseudonaja* species. It has been demonstrated in erabutoxin (a Type I α-ntx; uniprot: P60775) that mutations at position 29 have a drastic effect on the binding affinity of this toxin to the *Torpedo* nAChR [105]. Evaluation of mutations at the homologous site in α-cobratoxin (a Type II α-ntx; uniprot: P01391) were shown to have varied effects on binding affinities [106]. Although most sequences had a conserved phenylalanine residue at this position (F29; 49%), a number of others (33%) had tryptophan and histidine residues. It has been demonstrated that these substitutions (F29W and F29H) do not affect the binding affinity of Type II α-ntxs in any way [106]. Two other sequences (1%) had a leucine (F29L) residue in this position, which has been shown to substantially reduce the binding affinity of this toxin [106]. However, lysine residues in loop II (K23) and loop III (K49) have been shown to mediate the binding of Type II α-ntxs to α1-nAChRs and both these residues remain extremely well conserved in these toxins (Figure 7: Panel B; Supplementary Table 12). Thus, α1-nAChR targeting capability of Type II α-ntxs remains intact, despite the mutation at the 29th position. Similarly, it has been demonstrated that A28R mutation does not affect the function of Type II α-ntxs [107] and a large number of sequences (77%) at this site either had alanine or arginine residues. Mutations at R36 and F65 have shown to only slightly affect the binding capabilities of Type II α-ntxs at both neuronal and muscle nAChR. It should be noted that α-ntx possess several extremely well-conserved residues that mediate the specific binding of these toxins to either muscular or neuronal receptors (Figure 7). Thus, despite the mutations at R36 and F65, these toxins can still bind to muscular and neuronal receptors. These examples clearly depict how Type II α-ntxs cope with rapid mutation rate. R33 in Type II α-ntx has been shown to be the most important site for toxicity, as it caused the greatest effect on the function of Type II α-ntx in mutational studies. We noted that this site was extremely well conserved (86% identity; Supplementary Table 12). Mutational studies have also shown that T47 and D53, despite being very closely located to K49 on loop III, are unlikely to play a significant functional role [106]. Interestingly, our selection analyses have detected these sites as hypermutable, among 21 other hypermutable sites in Type II α-ntx (Figure 7: Panel B). Rare mutations at functional sites, such as I26R in erabutoxin (P60775), have been demonstrated to increase the affinity of α-ntx to their receptors [108]. Hence, rapidly evolving α-ntx could derive extremely useful functional variations by conserving most functional residues.

Most hypervariable regions (73%) in Type III α-ntx were concentrated in loop I and loop II (Figure 7), regions that harbour residues implicated in binding of Type I α-ntx to α_1_-nAChR (Supplementary Table 12). However, unlike Type I α-ntxs, Type III seem to have very well-conserved region between plesiotypic cysteines 9 and 10 [6], which form the 4th disulfide bond in both these toxins. The corresponding region in Type II α-ntx is also very well conserved, and shares with Type III α-ntx three highly conserved amino acids: S54, T55 and D56 (Figure 7). It is very likely that this region, being the only well-conserved region in Type III α-ntx, plays an important structural or functional role. The invariant Asp 56 in this region seems particularly important for the activity of this toxin clade. The observed pattern of accumulation of hypermutations (Figure 7) can also be explained by prey-specific and intraspecific conservation of residues in Type III α-ntx.

κ-bungarotoxins and cytotoxins were unique among all 3FTx lineages examined in this study, in having extremely conserved amino acid sequences (Figure 7 and Figure 8; Supplementary Figure 6). 52% (33 residues) and 65% (53 residues) of sites in κ-bungarotoxins and cytotoxins, respectively, were invariant. Although toxins from these two clades accumulated a few positively selected sites (5 and 2, respectively), they did not overlap with residues implicated in structural/functional stability. 

Mapping of hypermutable sites onto the alignments of the plesiotypic 3FTxs reveals a lack of conservation of most residues implicated in receptor binding of Type I and Type II elapid α-neurotoxins, which may be reflective of differences in taxon specificity. However, the residues implicated for activity in Type I and II may be specific to the assay utilised, as plesiotypic 3FTx have shown to be dramatically more potent against birds and/or lizards than mammals, but the specific receptors mediating the activity have not yet been elucidated. The residues implicated in Type I or Type II binding may also be inaccurate as a result of the former prevalence of fish assays (*Torpedo* species), which may not be reflective of residues actually important in binding to the receptors of natural prey items. 

The impact of the development of the venom-delivery system (VDS) and its subsequent specialisation in Caenophidia (advanced snakes) on the evolution of 3FTxs is intriguing. While 3FTx was recruited at the base of the snake evolutionary tree [9], only two NFF lineages (Colubridae and Psammophiinae) and one front-fanged lineage (Elapidae) express this toxin type as the dominant class [18]. As many ‘non-front-fanged’ advanced snakes use venom as part of a combined arsenal that includes variable use of constriction and bite pressure [67] there may therefore be less selective pressure on the diversification of 3FTx within most members of the NFF lineages. Viperids, on the other hand, rely on venom for prey subjugation, and possess arguably the most sophisticated venom-delivery apparatus found amongst snakes (hinged hollow fangs). Viperid snakes utilise a primarily haemolytic and necrotic venom arsenal for prey subjugation and thus 3FTx have not diversified greatly within this family. This may be linked to prey preference, particularly in pit vipers, which possess heat-sensing pits for targeting endothermic (“warm-blooded”) prey. As plesiotypic 3FTx have been shown to be more toxic to birds and reptiles than mammals [21,109], snakes that specialise in feeding on mammals may have less use for this toxin type. Conversely, the earliest elapid snake lineages, with hollow, fixed front fangs, may have fed primarily on reptiles as plesiomorphic elapid snakes do today (e.g., [83,85]), and employed 3FTx as their primary weapon. Hence, three-finger toxins in these snakes underwent rapid and explosive diversification under the significant influence of positive Darwinian selection, resulting in the formation of a myriad of three-finger toxin forms which appear to be exclusive to the elapid snakes (Figure 1). Interestingly, this diversification of 3FTx within elapids led to the origin of new forms that were highly potent against mammals, facilitating more efficient predation on a wider range of prey species including the rodent prey specialised upon by some extant lineages of elapids such as *Oxyuranus* and *Pseudonaja*. The plesiotypic 3FTx, which are expressed in relatively lower amounts in elapids, viperids and a majority of “non-front-fanged” advanced snakes (except in Colubridae and Psammophiinae venoms where they are the dominant toxin types), could still play an ancillary envenoming function and thus still be subject to RAVER, potentially facilitating the apotyposis of novel functional forms within this clade. Further sequencing and biochemical activity testing of plesiotypic 3FTx genes will unravel their molecular evolutionary history and the underlying mechanisms of action.

## 4. Conclusion

In this study, we investigated the following hypotheses: (i) that 3FTx with specific sites of action are involved in coevolutionary arms races with receptors of prey species and thus are evolving under the influence of positive selection; (ii) that cytotoxic 3FTx, which interact non-specifically with cell membranes, are not involved in chemical arms race, and thus are constrained by negative selection; (iii) that, in venoms in which 3FTx are a major component (Elapidae, Colubridae and Psammophiinae), 3FTx are rapidly evolving under positive selection (with the exception of cytotoxic 3FTx in the venom of elapid snakes); (iv) that in venoms in which 3FTx are a minor component (Viperidae), 3FTx evolve under a neutral selection regime and do not experience positive selection; and (v) the evolution of the advanced venom delivery system in elapid snakes led to an increase in positive selection pressure on 3FTx, resulting in the diversity of functional forms present in the venoms of snakes from this family. We were able to confirm hypotheses i, ii, iii and v, but surprisingly the viperid 3FTx were unravelled to have evolved under the influence of positive selection, resulting in the rejection of hypothesis iv. 

In addition, we found that point mutations, coupled with focal mutagenesis and positive selection, have played a primary role in the evolution and diversification of snake venom three-finger toxins. We thus reject ASSET as a viable theory for explaining the molecular evolution of three-finger toxins. Instead, we propose “Rapid Accumulation of Variations in Exposed Residues” (RAVER) to explain the dynamic evolution of 3FTx. We elucidate an exquisite molecular mechanism in which the molecular surface, and the regions not implicated in structural/functional stability rapidly diversify, while the core structural/functional residues remain largely constrained by negative selection. However, even these highly conserved residues occasionally accumulate point mutations, especially in toxins with multiple targets such as Type II α-ntx. Rare mutations in these highly conserved residues can generate unique toxin forms with novel folds, which might be more potent than the plesiotypic form. 

We have also shown for the first time that not all 3FTx forms are influenced by positive selection and highlighted the differential rate of evolution of various 3FTx types. As well as confirming hypothesis ii, by showing that cytotoxins have been constrained by negative selection, we found that unique forms like the κ-ntx have experienced weaker influence of positive selection than the α-ntx.

These results underscore the dynamic nature of venom evolution, where a myriad of forces, such as morphological refinement of the venom delivery system, shifts in feeding ecology, coevolutionary chemical arms races driving toxin neofunctionalisation via changes in targeting and evolution of toxin complexes with multiple subunits. all influence the molecular evolutionary history of toxins. This generates a molecular complexity that is both a blessing and a curse. On one hand, this complexity complicates snakebite treatment because of highly variable clinical sequelae and relative cross-reactivity of available antivenoms. On the other hand, it provides a rich pool of novel investigational ligands with tremendous potential for biodiscovery. 

It should be emphasised that this study only focused upon structural/functional 3FTx variants for which there were sufficient nucleotides to analyse. As shown in Figure 1 and Table 1, there are numerous functional variants that were not studied due to insufficient nucleotide sequences available (with some known only from peptide sequences). In addition, as is clearly evident in Figure 1 and has been covered in-depth by us previously [6], there is a tremendous diversity of 3FTxs for which no functional activity has been determined. Thus, 3FTx represent a tremendous resource not only for investigating the fundamentals of molecular evolution but also as a vast pool of novel peptides for biodiscovery. It is hoped that this study will stimulate further work on this very interesting class of snake toxin.

## 5. Materials and Methods

### 5.1. Specimens

*Azemiops feae* specimen was from Hunan, China. 

### 5.2. Azemiops Feae Library Construction

Total RNA was extracted from the dissected venom gland using the standard TRIzol Plus method (Invitrogen). Extracts were enriched for mRNA using standard RNeasy mRNA mini kit (Qiagen) protocol. mRNA was reverse transcribed, fragmented and ligated to a unique 10-base multiplex identifier (MID) tag prepared using standard protocols and applied to one PicoTitrePlate (PTP) for simultaneous amplification and sequencing on a Roche 454 GS FLX+ Titanium platform (Australian Genome Research Facility). Automated grouping and analysis of sample-specific MID reads informatically separated sequences from the other transcriptomes on the plates, which were then post-processed to remove low quality sequences before de novo assembly into contiguous sequences (contigs) using v 3.4.0.1 of the MIRA software program, resulting in 1656 contigs from 811,967 assembled bases (490 average per contig). Assembled contigs were processed using CLC Main Work Bench (CLC-Bio) and Blast2GO bioinformatic suite [110] to provide Gene Ontology, BLAST and domain/Interpro annotation.

### 5.3. Sequence Retrieval and Alignment

To assess the molecular evolutionary history of various three-finger toxins, we retrieved nucleotide sequences from National Center for Biotechnology Information [111]. The new toxin sequence recovered from *Azemiops feae* in this study was identified by comparison of the translated DNA sequence with those of previously characterised toxins using a BLAST search [112] implemented in the UniProtKB protein database. Translated nucleotide sequences were aligned using MUSCLE 3.8 [113] and the alignments were manually inspected to rectify errors. Sequences were refined to remove regions with gaps in more than 50% of the sequences. All alignments are available as supplementary file 1, while all the nucleotide sequences (with their NCBI accession codes) analysed in this study are deposited as supplementary file 2.

### 5.4. Phylogenetics

Phylogenetic analyses were performed to allow the reconstruction of the molecular evolutionary history of various types of three-finger toxins. Since many three-finger toxin forms are only known from their amino acid sequences, amino acid datasets were used for the Bayesian inference. Trees were generated using both Bayesian and maximum-likelihood inferences. MrBayes 3.2.1 [114] was employed for the Bayesian inference, using lset rates = invgamma with prset aamodelpr = mixed command, which enables the program to optimise between nine different amino acid substitution matrices implemented in MrBayes. WAG [115] was chosen as the best substitution matrix by the program. The analysis was performed by running a minimum of 1×10^7^ generations in four chains, and saving every 100th tree. The log-likelihood score of each saved tree was plotted against the number of generations to establish the point at which the log likelihood scores reached their asymptote, and the posterior probabilities for clades established by constructing a majority-rule consensus tree for all trees generated after completion of the burn-in phase. Maximum-likelihood method implemented in PhyML [116] was also employed on the nucleotide datasets and node support was evaluated with 1,000 bootstrapping replicates.

### 5.5. Test for Recombination

To overcome the effects of recombination on the phylogenetic and evolutionary interpretations [117], we employed Single Breakpoint algorithms implemented in the HyPhy package and assessed recombination on all the toxin forms examined in this study [118,119]. When potential breakpoints were detected using the small sample Akaike information Criterion (AICc), the multiple sequence alignments were compartmentalised before conducting selection analyses, so as to allow the different recombinant regions to have distinct phylogenetic topology.

### 5.6. Selection Analyses

We evaluated the influence of natural selection on various types of three-finger toxins using maximum-likelihood models [120,121] implemented in Codeml of the PAML package [122]. We utilised the three-ratio model as well as the optimised branch-site test [123,124] to detect evolutionary selection pressures acting upon various toxin lineages. The three-ratio model evaluates selection across the lineages alone, while the branch-site model allows *ω* to vary across the sites of the protein and along the branches in the tree. The latter is known for its reasonable power and accuracy to detect short bursts of episodic adaptations [123]. However, both the three-ratio and branch-site models require the foreground (lineages under positive selection) and background lineages (lineages lacking positive selection) to be defined *a priori*. Such predefined biological hypotheses are often unavailable and it becomes difficult to define the foreground lineages. Therefore, we treated each 3FTx type being compared as a foreground branch alternatively and tested multiple hypotheses. A likelihood-ratio test was then conducted by comparing the model that allows ω to be greater than 1 in the foreground branch, with a null model that does not (*ω* constrained 1). It has been suggested that while implementing multiple hypotheses using branch and branch-site models, it is necessary to control the family-wise error rate (FWER or Type I error) [123]. We used Bonferroni correction to account for such errors. It uses α/n as the significance level to test each hypothesis, where “α” is the significance level and “n” is the number of independent true null hypotheses. We further utilised branch-site Random Effects Likelihood (REL) [63] to identify lineages evolving under the influence of episodic diversifying selection pressures. Unlike the aforementioned lineage-specific models, branch-site REL does not require *a priori* identification of foreground and background branches. Moreover, branch-site tests have been shown to be prone to false positives when the background lineages are also under the influence of positive selection and branch-site test was designed to address these shortcomings [63].

Since lineage-specific models assess the effects of selection only along the branches, they often fail to identify regions in proteins that might be affected by episodic selection pressures and ultimately underestimate the strength of selection. Hence, we employed site-specific models that estimate positive selection statistically as a non-synonymous-to-synonymous nucleotide-substitution rate ratio (*ω*) significantly greater than 1. We compared likelihood values for three pairs of models with different assumed ω distributions as no *a priori* expectation exists for the same: M0 (constant ω rates across all sites) *versus* M3 (allows ω to vary across sites within “*n*” discrete categories, *n* ≥ 3); M1a (a model of neutral evolution) where all sites are assumed to be either under negative (*ω* < 1) or neutral selection (*ω* = 1) *versus* M2a (a model of positive selection) which in addition to the site classes mentioned for M1a, assumes a third category of sites; sites with *ω* > 1 (positive selection) and M7 (Beta) *versus* M8 (Beta and ω), and models that mirror the evolutionary constraints of M1 and M2 but assume that *ω* values are drawn from a beta distribution [125]. Only if the alternative models (M3, M2a and M8: allow sites with *ω* > 1) show a better fit in Likelihood Ratio Test (LRT) relative to their null models (M0, M1a and M7: do not allow sites *ω* > 1), are their results considered significant. It should be noted that although we have reported the results of all site-specific models (M2a, M3 and M8), only the results of M8, the most accurate model among these, were considered for downstream analyses (Figure 3, Figure 4, Figure 5, Figure 6, Figure 7, and Figure 8; Table 4 and Table 5). LRT is estimated as twice the difference in maximum likelihood values between nested models and compared with the χ^2^ distribution with the appropriate degree of freedom—the difference in the number of parameters between the two models. The Bayes empirical Bayes (BEB) approach [126] was used to identify amino acids under positive selection by calculating the posterior probabilities that a particular amino acid belongs to a given selection class (neutral, conserved or highly variable). Sites with greater posterior probability (*PP* ≥ 95%) of belonging to the “*ω* > 1 class” were inferred to be positively selected.

FUBAR [127] implemented in HyPhy [128] was employed to detect codon sites evolving under the influence of pervasive diversifying and purifying selection pressures. The Mixed Effects Model of Evolution (MEME) [129] was also employed to efficiently detect sites that experience diversifying selection for a short period in the evolutionary timescale. Non-synonymous mutations that introduce extremely variant amino acids are more likely to influence the structure-function, and hence the fitness of the organism, relative to mutations that introduce amino acids with similar side-chains as the ancestral residues. Further support for the results of the nucleotide-level selection analyses was obtained and the radicalness of mutations were assessed using a complementary protein-level approach implemented in TreeSAAP [130].

Direct comparison of *ω* values computed from different datasets can be misleading, as they can have different proportions of sites under selection. Hence, we assessed the selection pressures shaping different 3FTx clades by employing clade model analyses implemented in Codeml and simultaneously estimated ω values [131]. The significance of the analysis was tested by comparing the likelihood of this model with that of model M1a. To clearly depict the proportion of sites under different regimes of selection, an evolutionary fingerprint analysis was carried out using the evolutionary selection distance (ESD) algorithm implemented in datamonkey [132].

### 5.7. Structural Analyses

To depict the natural selection pressures influencing the evolution of various three-finger toxins, we mapped the sites under positive selection on the homology models created using the Phyre 2 webserver [133]. Pymol 1.3 [134] was used to visualise and generate the images of homology models. The Consurf webserver [135] was used for mapping the evolutionary selection pressures on the three-dimensional homology models. GETAREA [136] was used to calculate the Accessible Surface Area (ASA) or the solvent exposure of amino-acid side chains. It uses the atom co-ordinates of the PDB file and indicates if a residue is buried or exposed to the surrounding medium by comparing the ratio between side chain ASA and the “random coil” values per residue. An amino acid is considered to be buried if it has an ASA less than 20% and exposed if the ASA is more than or equal to 50%. When ASA ratio lies between 40% and 50%, it is highly likely that the residues have their side chains exposed to the surrounding medium.

## Supplementary Files

Supplementary File 1 Supplementary (ZIP, 3550 KB)
