# Supplementary material for: Three-Fingered RAVERs: Rapid Accumulation of Variations in Exposed Residues of Snake Venom Toxins
Source: Toxins (Basel). 2013 Nov 18;5(11):2172–208. doi: 10.3390/toxins5112172 (PMC3847720; doi:10.3390/toxins5112172)
Supplement: Supplementary File 1 — Supplementary (ZIP, 3550 KB) [file toxins-05-02172-s001.zip › toxins-42720_supplementary material/Supplementary Figure 1.pdf]

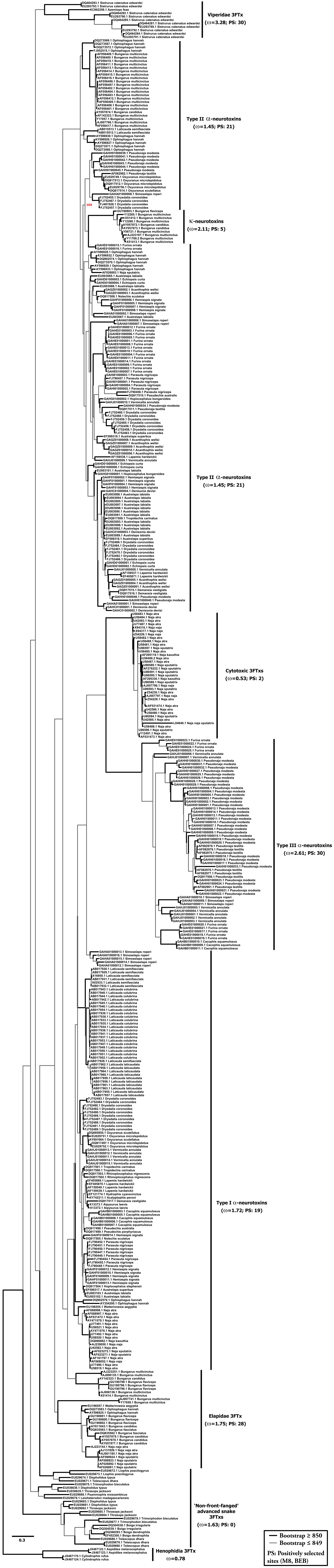

Viperidae 3FTx
(ω=3.28; PS: 30)
Type II α-neurotoxins
(ω=1.45; PS: 21)
κ-neurotoxins
(ω=2.11; PS: 5)
Type II α-neurotoxins
(ω=1.45; PS: 21)
Cytotoxic 3FTxs
(ω=0.53; PS: 2)
Type III α-neurotoxins
(ω=2.61; PS: 30)
Type I α-neurotoxins
(ω=1.72; PS: 19)
Elapidae 3FTx
(ω=1.75; PS: 28)
'Non-front-fanged' advanced snake 3FTxs
(ω=1.63; PS: 0)
Henophidia 3FTx
ω=0.78
0.3
Bootstrap ≥ 850
Bootstrap ≤ 849
PS: Positively selected sites (M8, BEB)

A maximum-likelihood tree (model GTR + I + G; ln L = -21068.86248; Best of NNIs and SPRs) of three-finger toxins is presented here. Node support was evaluated through 1000 bootstrap replicates. Branches with node support of more than 850/1000 replicates are indicated as thick lines. Site-model 8 computed omega and the total number of positively selected sites detected by its Bayes Empirical Bayes (BEB) approach (PP $\geq$ 0.95) are also indicated for each toxin clade.
