# Supplementary material for: Three-Fingered RAVERs: Rapid Accumulation of Variations in Exposed Residues of Snake Venom Toxins
Source: Toxins (Basel). 2013 Nov 18;5(11):2172–208. doi: 10.3390/toxins5112172 (PMC3847720; doi:10.3390/toxins5112172)
Supplement: Supplementary File 1 — Supplementary (ZIP, 3550 KB) [file toxins-05-02172-s001.zip › toxins-42720_supplementary material/Supplementary Tables 1-13.pdf]

Table 1. Maximum-likelihood parameter estimates for the plesiotypic 3FTX from 'non-front-fanged' advanced snakes

Model	Likelihood (ι)	ω_0[a]	Parameters	Sign.[b]	No. of Sites with $\omega > 1$[c]
					B.E.B
**M0 (One ratio)**	-3678.383646	1.29	$= \omega_0$		-
**M1 (Neutral)**	-3585.956425	0.72	P_0: 0.314<br>ω_0: 0.126<br>P_1: 0.685<br>ω1:1.0		-
**M2 (Selection)***	-3540.037278	1.71	P_0: 0.218<br>ω_0: 0.13<br>P_1: 0.328<br>ω_1:1.0<br>P_2: 0.452<br>ω_2: 3.0	$P << 0.001$	18 ($PP \geq 0.99$)<br>8 ($P \geq 0.95$)
**M3 (Discrete)***	-3539.925471	1.67	P_0: 0.206<br>ω_0: 0.11<br>P_1: 0.330<br>ω_1: 0.91<br>P_2: 0.462<br>ω_2: 2.91	$P << 0.001$	-
**M7 (beta)**	-3588.471551	0.71	p: 0.34252<br>q: 0.13966		-
**M8 (beta and ω)***	-3539.994764	1.63	p_0: 0.534<br>p: 0.447<br>q: 0.316<br>p1: 0.465<br>ω: 2.84	$P << 0.001$	27 ($PP \geq 0.99$)<br>10 ($P > 0.95$)

Legend:

a: dn/ds (weighted average)

b: Significance of the model in comparison with the null model

c: Number of sites with $\omega > 1$ under the Bayes empirical Bayes approach with a posterior probability (PP) more than or equal to 0.99 and 0.95

* Models which allow $\omega > 1$

Table 2. Maximum-likelihood parameter estimates for the plesiotypic 3FTX from Viperidae

Model	Likelihood (ι)	ω_0[a]	Parameters	Sign.[b]	No. of Sites with $\omega > 1$[c]
					**B.E.B**
**M0 (One ratio)**	-1195.940485	1.79	$= \omega_0$		-
**M1 (Neutral)**	-1178.683529	0.67	P_0: 0.344<br>ω_0: 0.05<br>P_1: 0.655<br>ω1: 1.0		-
**M2 (Selection)***	-1155.587491	3.28	P_0: 0.454<br>ω_0: 0.37<br>P_1: 0.0<br>ω_1:1.0<br>P_2: 0.545<br>ω_2: 5.70	P << 0.001	13 (PP ≥ 0.99)<br>11 (P ≥ 0.95)
**M3 (Discrete)***	-1153.672936	3.93	P_0: 0.427<br>ω_0: 0.33<br>P_1: 0.485<br>ω_1: 4.86<br>P_2: 0.086<br>ω_2: 16.4	P << 0.001	-
**M7 (beta)**	-1179.346712	0.71	p: 0.03813<br>q: 0.01410		-
**M8 (beta and ω)***	-1155.588330	3.28	p_0: 0.454<br>p: 58.52<br>q: 99.0<br>p1: 0.545<br>ω: 5.71	P << 0.001	18 (PP ≥ 0.99)<br>12 (P > 0.95)

Legend:

a: dn/ds (weighted average)

b: Significance of the model in comparison with the null model

c: Number of sites with $\omega > 1$ under the Bayes empirical Bayes approach with a posterior probability (PP) more than or equal to 0.99 and 0.95

* Models which allow $\omega > 1$

Table 3. Maximum-likelihood parameter estimates for the plesiotypic 3FTX from Elapidae

Model	Likelihood (ι)	ω_0[a]	Parameters	Sign.[b]	No. of Sites with $\omega > 1$[c]
					**B.E.B**
**M0 (One ratio)**	-1885.178988	1.30	$= \omega_0$		-
**M1 (Neutral)**	-1833.771080	0.60	P_0: 0.419<br>ω_0: 0.05<br>P_1: 0.580<br>ω1: 1.0		-
**M2 (Selection)***	-1807.546142	1.79	P_0: 0.355<br>ω_0: 0.08<br>P_1: 0.249<br>ω_1:1.0<br>P_2: 0.395<br>ω_2: 3.83	$P << 0.001$	15 ($PP \geq 0.99$)<br>9 ($P \geq 0.95$)
**M3 (Discrete)***	-1807.476797	1.75	P_0: 0.313<br>ω_0: 0.24<br>P_1: 0.279<br>ω_1: 0.81<br>P_2: 0.407<br>ω_2: 3.70	$P << 0.001$	-
**M7 (beta)**	-1835.536623	0.61	p: 0.03086<br>q: 0.01787		-
**M8 (beta and ω)***	-1807.523245	1.75	p_0: 0.594<br>p: 0.236<br>q: 0.325<br>p1: 0.405<br>ω: 3.72	$P << 0.001$	22 ($PP \geq 0.99$)<br>6 ($P > 0.95$)

Legend:

a: dn/ds (weighted average)

b: Significance of the model in comparison with the null model

c: Number of sites with $\omega > 1$ under the Bayes empirical Bayes approach with a posterior probability (PP) more than or equal to 0.99 and 0.95

* Models which allow $\omega > 1$

Table 4. Maximum-likelihood parameter estimates for type I (short-chain) α-neurotoxins

Model	Likelihood (ι)	ω_0[a]	Parameters	Sign.[b]	No. of Sites with ω > 1[c]
					**B.E.B**
**M0 (One ratio)**	-3446.886907	1.92	$= \omega_0$		-
**M1 (Neutral)**	-3372.647732	0.57	P_0: 0.485<br>ω_0: 0.12<br>P_1: 0.514<br>ω1: 1.0		-
**M2 (Selection)***	-3285.128559	1.61	P_0: 0.349<br>ω_0: 0.16<br>P_1: 0.369<br>ω_1:1.0<br>P_2: 0.280<br>ω_2: 4.24	P << 0.001	11 (PP ≥ 0.99)<br>2 (P > 0.95)
**M3 (Discrete)***	-3264.912926	2.16	P_0: 0.487<br>ω_0: 0.33<br>P_1: 0.410<br>ω_1: 2.67<br>P_2: 0.102<br>ω_2: 8.86	P << 0.001	-
**M7 (beta)**	-3380.125103	0.57	p:0.28637<br>q:0.21125		-
**M8 (beta and ω)***	-3287.175727	1.72	p_0: 0.703<br>p: 0.303<br>q: 0.193<br>p1: 0.296<br>ω: 4.35	P << 0.001	13 (PP ≥ 0.99)<br>6 (P > 0.95)

Legend:

a: dn/ds (weighted average)

b: Significance of the model in comparison with the null model

c: Number of sites with ω > 1 under the Bayes empirical Bayes approach with a posterior probability (PP) more than or equal to 0.99 and 0.95

* Models which allow ω > 1

Table 5. Maximum-likelihood parameter estimates for type II (long-chain) α-neurotoxins

Model	Likelihood (ι)	ω_0 [a]	Parameters	Sign. [b]	No. of Sites with ω > 1 [c]
					**B.E.B**
**M0 (One ratio)**	-7338.701541	2.01	$= \omega_0$		-
**M1 (Neutral)**	-7080.814722	0.62	P_0: 0.405<br>ω_0: 0.06<br>P_1: 0.594<br>ω1: 1.0		-
**M2 (Selection)***	-6845.160604	1.43	P_0: 0.384<br>ω_0: 0.10<br>P_1: 0.356<br>ω_1:1.0<br>P_2: 0.259<br>ω_2: 4.008	P << 0.001	17 (PP ≥ 0.99)<br>2 (P > 0.95)
**M3 (Discrete)***	-6801.590700	2.37	P_0: 0.402<br>ω_0: 0.18<br>P_1: 0.461<br>ω_1: 2.54<br>P_2: 0.136<br>ω_2: 8.28	P << 0.001	-
**M7 (beta)**	-7078.743858	0.53	p: 0.18047<br>q: 0.16001		-
**M8 (beta and ω)***	-6853.164183	1.45	p_0: 0.736<br>p: 0.210<br>q: 0.168<br>p1: 0.263<br>ω: 3.97	P << 0.001	19 (PP ≥ 0.99)<br>2 (P > 0.95)

Legend:

a: dn/ds (weighted average)

b: Significance of the model in comparison with the null model

c: Number of sites with ω > 1 under the Bayes empirical Bayes approach with a posterior probability (PP) more than or equal to 0.99 and 0.95

* Models which allow ω > 1

Table 6. Maximum-likelihood parameter estimates for type III α-neurotoxins

Model	Likelihood (ι)	ω_0[a]	Parameters	Sign.[b]	No. of Sites with $\omega > 1$[c]
					**B.E.B**
**M0 (One ratio)**	-3312.727326	2.59	$= \omega_0$		-
**M1 (Neutral)**	-3210.492742	0.58	P_0: 0.428<br>ω_0: 0.04<br>P_1: 0.571<br>ω_1: 1.0		-
**M2 (Selection)***	-3089.176251	2.59	P_0: 0.333<br>ω_0: 0.03<br>P_1: 0.247<br>ω_1:1.0<br>P_2: 0.418<br>ω_2: 5.57	P << 0.001	26 (PP ≥ 0.99)<br>1 (P > 0.95)
**M3 (Discrete)***	-3079.356207	3.27	P_0: 0.413<br>ω_0: 0.12<br>P_1: 0.272<br>ω_1: 2.58<br>P_2: 0.314<br>ω_2: 8.0	P << 0.001	-
**M7 (beta)**	-3215.119806	0.42	p: 0.01764<br>q: 0.02272		-
**M8 (beta and ω)***	-3089.367982	2.61	p_0: 0.577<br>p: 0.019<br>q: 0.025<br>p1: 0.422<br>ω: 5.61	P << 0.001	26 (PP ≥ 0.99)<br>4 (P > 0.95)

Legend:

a: dn/ds (weighted average)

b: Significance of the model in comparison with the null model

c: Number of sites with $\omega > 1$ under the Bayes empirical Bayes approach with a posterior probability (PP) more than or equal to 0.99 and 0.95

* Models which allow $\omega > 1$

Table 7. Maximum-likelihood parameter estimates for *Oxyuranus* / *Pseudonaja* Type II (long-chain) α-neurotoxin with cysteine doublet

Model	Likelihood (ι)	ω_0[a]	Parameters	Sign.[b]	No. of Sites with $\omega > 1$[c]
					**B.E.B**
**M0 (One ratio)**	-953.657356	0.97	$= \omega_0$		-
**M1 (Neutral)**	-943.204575	0.62	P_0: 0.375<br>ω_0: 0.0<br>P_1: 0.624<br>ω1: 1.0		-
**M2 (Selection)***	-918.331297	3.57	P_0: 0.157<br>ω_0: 0.0<br>P_1: 0.529<br>ω_1: 1.0<br>P_2: 0.312<br>ω_2: 9.75	P << 0.001	10 (PP ≥ 0.99)<br>7 (P > 0.95)
**M3 (Discrete)***	-916.570131	4.82	P_0: 0.625<br>ω_0: 0.62<br>P_1: 0.341<br>ω_1: 8.51<br>P_2: 0.032<br>ω_2: 47.15	P << 0.001	-
**M7 (beta)**	-943.258977	0.60	p: 0.00754<br>q: 0.00500		-
**M8 (beta and ω)***	-918.427424	3.67	p_0: 0.674<br>p: 17.55<br>q: 6.79<br>p_1: 0.325<br>ω: 9.77	P << 0.001	11(PP ≥ 0.99)<br>7 (P > 0.95)

Legend:

a: dn/ds (weighted average)

b: Significance of the model in comparison with the null model

c: Number of sites with $\omega > 1$ under the Bayes empirical Bayes approach with a posterior probability (PP) more than or equal to 0.99 and 0.95

* Models which allow $\omega > 1$

Table 8. Maximum-likelihood parameter estimates for *Oxyuranus* / *Pseudonaja* Type II (long-chain) α-neurotoxin without cysteine doublet

Model	Likelihood (ι)	ω_0[a]	Parameters	Sign.[b]	No. of Sites with ω > 1[c]
					**B.E.B**
**M0 (One ratio)**	-843.272386	2.69	$= \omega_0$		-
**M1 (Neutral)**	-845.722868	0.76	P_0: 0.237<br>ω_0: 0.0<br>P_1: 0.762<br>ω1: 1.0		-
**M2 (Selection)***	-832.929158	3.41	P_0: 0.416<br>ω_0: 0.06<br>P_1: 0.0<br>ω_1:1.0<br>P_2: 0.583<br>ω_2: 5.80	P << 0.001	2 (PP ≥ 0.99)<br>12 (P > 0.95)
**M3 (Discrete)***	-832.929158	3.41	P_0: 0.416<br>ω_0: 0.06<br>P_1: 0.476<br>ω_1: 5.80<br>P_2: 0.107<br>ω_2: 5.80	P << 0.001	-
**M7 (beta)**	-846.563004	0.90	p: 0.04683<br>q: 0.00500		-
**M8 (beta and ω)***	-832.929285	3.41	p_0: 0.416<br>p: 6.49<br>q: 98.96<br>p1: 0.583<br>ω: 5.80	P << 0.001	9 (PP ≥ 0.99)<br>11 (P > 0.95)

Legend:

a: dn/ds (weighted average)

b: Significance of the model in comparison with the null model

c: Number of sites with ω > 1 under the Bayes empirical Bayes approach with a posterior probability (PP) more than or equal to 0.99 and 0.95

* Models which allow ω > 1

Table 9. Maximum-likelihood parameter estimates for kappa three-finger toxins

Model	Likelihood (ι)	ω_0[a]	Parameters	Sign.[b]	No. of Sites with $\omega > 1$[c]
					**B.E.B**
**M0 (One ratio)**	-745.132731	1.64	$= \omega_0$		-
**M1 (Neutral)**	-743.698028	0.66	P_0: 0.344<br>ω_0: 0.03<br>P_1: 0.655<br>ω_1: 1.0		-
**M2 (Selection)***	-728.968534	2.11	P_0: 0.782<br>ω_0: 1.0<br>P_1: 0.124<br>ω_1:1.0<br>P_2: 0.09<br>ω_2: 12.96	P << 0.001	3 (PP ≥ 0.99)<br>2 (P > 0.95)
**M3 (Discrete)***	-728.457588	2.30	P_0: 0.871<br>ω_0: 0.95<br>P_1: 0.1<br>ω_1: 7.61<br>P_2: 0.2<br>ω_2: 25.61	P << 0.001	-
**M7 (beta)**	-743.727286	0.70	p: 0.01191<br>q: 0.00500		-
**M8 (beta and ω)***	-728.968534	2.11	p_0: 0.906<br>p: 3.212<br>q: 0.005<br>p1: 0.093<br>ω: 12.96	P << 0.001	3 (PP ≥ 0.99)<br>2 (P > 0.95)

Legend:

a: dn/ds (weighted average)

b: Significance of the model in comparison with the null model

c: Number of sites with $\omega > 1$ under the Bayes empirical Bayes approach with a posterior probability (PP) more than or equal to 0.99 and 0.95

* Models which allow $\omega > 1$

Table 10. Maximum-likelihood parameter estimates for cytotoxic three-finger toxins

Model	Likelihood (ι)	ω_0[a]	Parameters	Sign.[b]	No. of Sites with $\omega > 1$[c]
					**B.E.B**
**M0 (One ratio)**	-970.316160	0.32	$= \omega_0$		-
**M1 (Neutral)**	-952.107186	0.33	P_0: 0.712<br>ω_0: 0.06<br>P_1: 0.287<br>ω1: 1.0		-
**M2 (Selection)***	-945.995693	0.57	P_0: 0.675<br>ω_0: 0.06<br>P_1: 0.294<br>ω_1:1.0<br>P_2: 0.029<br>ω_2: 8.18	$P << 0.001$	1 ($PP \geq 0.99$)<br>1 ($P \geq 0.95$)
**M3 (Discrete)***	-945.501674	0.52	P_0: 0.518<br>ω_0: 0.0<br>P_1: 0.446<br>ω_1: 0.62<br>P_2: 0.034<br>ω_2: 7.02	$P < 0.01$	-
**M7 (beta)**	-952.219533	0.31	p: 0.13885<br>q: 0.29569		-
**M8 (beta and ω)***	-945.530089	0.53	p_0: 0.969<br>p: 0.173<br>q: 0.387<br>p1: 0.03<br>ω: 7.53	$P < 0.01$	2 ($PP \geq 0.99$)<br>0 ($P > 0.95$)

Legend:

a: dn/ds (weighted average)

b: Significance of the model in comparison with the null model

c: Number of sites with $\omega > 1$ under the Bayes empirical Bayes approach with a posterior probability (PP) more than or equal to 0.99 and 0.95

* Models which allow $\omega > 1$

$p > 0.05^{N.S}$: Not significant at 0.05

Supplementary Table 11. Nucleotide and complementary amino acid-level selection assessment

Site		CodeML		TreeSAAP		ASA
Codon	AA	M2a[a]	M8[b]	Property[c]	Magnitude[d]	
**Type I α neurotoxins**						
22	M	4.428±0.412 (0.989)*	4.456±0.247 (0.998)**	–	–	35.5 NA
**37**	**M**	**4.466±0.209 (1.0)****	**4.461±0.201 (1.0)****	**α*c***	**6**	**39.9 Part. Exposed**
**39**	**A**	**4.444±0.341 (0.994)****	**4.456±0.243 (0.999)****	**α*c***	**6**	**95.5 Exposed**
**40**	**-**	**4.290±0.783 (0.950**	**4.407±0.479 (0.985)***	**α*c***	**6**	**100 Exposed**
**43**	**S**	**4.274±0.813 (0.945)**	**4.424±0.411 (0.990)***	***Hnc*, α*c***	**7, 6**	**69.5 Exposed**
**49**	**T**	**4.466±0.208 (1.0)****	**4.461±0.201 (1.0)****	***Hnc*, α*c***	**7, 6**	**66.4 Exposed**
**51**	**R**	**4.466±0.208 (1.0)****	**4.461±0.201 (1.0)****	***Hnc*, R_α**	**8, 6**	**96.4 Exposed**
**53**	**H**	**4.466±0.210 (1.0)****	**4.461±0.202 (1.0)****	***Hnc*, R_α**	**8, 6**	**90.2 Exposed**
**56**	**T**	**4.224±0.900 (0.931)**	**4.403±0.493 (0.984)***	***Hnc*, α*c*, R_α**	**8, 8, 6**	**77.9 Exposed**
**57**	**I**	**4.428±0.414 (0.989)***	**4.455±0.253 (0.998)****	***Hnc*, α*c*, R_α**	**8, 8, 6**	**72.6 Exposed**
**58**	**I**	**4.147±1.014 (0.909)**	**4.392±0.527 (0.981)***	***Hnc*, α*c*, R_α**	**8, 8, 6**	**8.7 Buried**
**66**	**K**	**4.466±0.208 (1.0)****	**4.461±0.201 (1.0)****	***Hnc*, α*c*, R_α**	**8, 6, 6**	**40.5 Part. Exposed**
**69**	**P**	**4.466±0.208 (1.0)****	**4.461±0.201 (1.0)****	***Hnc*, α*c*, R_α**	**8, 6, 6**	**99.7 Exposed**
**70**	**G**	**4.455±0.283 (0.997)****	**4.459±0.219 (0.999)****	***Hnc*, α*c*, R_α**	**8, 7, 6**	**98 Exposed**
**71**	**I**	**3.812±1.360 (0.814)**	**4.289±0.782 (0.952)***	**α*c***	**7**	**23.5 NA**
**72**	**K**	**4.466±0.208 (1.0)****	**4.461±0.201 (1.0)****	**α*c***	**7**	**65.9 Exposed**
**73**	**L**	**3.990±1.202 (0.864)**	**4.310±0.741 (0.958)***	**α*c***	**7**	**14.2 Buried**
**74**	**E**	**4.466±0.208 (1.0)****	**4.461±0.201 (1.0)****	***Hnc*, α*c***	**7, 7**	**69.8 Exposed**
**77**	**K**	**4.466±0.208 (1.0)****	**4.461±0.201 (1.0)****	***Hnc*, α*c***	**7, 7**	**41.3 Part. Exposed**
**Type II α neurotoxins**						
**22**	**L**	**4.248±0.434 (1.0)****	**3.612±0.649 (0.965)***	***h***	**6**	**17.7 Buried**

Site		CodeML		TreeSAAP		ASA
26	M	4.248±0.434<br>(1.0)**	3.735±0.424<br>(1.0)**	*h*	6	0<br>Buried
29	P	4.248±0.434<br>(1.0)**	3.735±0.424<br>(1.0)**	*h*	6	100<br>Exposed
30	K	4.235±0.477<br>(0.996)**	3.733±0.428<br>(1.0)**	*h*	6	46<br>Part. Exposed
31	T	4.248±0.434<br>(1.0)**	3.735±0.424<br>(1.0)**	*h*	6	38.3<br>NA
33	R	4.248±0.434<br>(1.0)**	3.735±0.424<br>(1.0)**	*h*	6	45.7<br>Part. Exposed
39	E	4.217±0.529<br>(0.991)**	3.730±0.434<br>(0.999)**	*pK', h*	7, 6	62.4<br>Exposed
40	N	4.247±0.436<br>(1.0)**	3.735±0.424<br>(1.0)**	*pK', h*	7, 6	70.4<br>Exposed
41	L	4.245±0.445<br>(0.999)**	3.734±0.426<br>(1.0)**	*pK', h*	7, 6	18.4<br>Buried
50	P	4.248±0.434<br>(1.0)**	3.735±0.424<br>(1.0)**	*pK', h*	8, 8	57.7<br>Exposed
51	R	4.248±0.434<br>(1.0)**	3.735±0.424<br>(1.0)**	*pK', h*	8, 8	50.9<br>Exposed
53	S	4.248±0.434<br>(1.0)**	3.735±0.424<br>(1.0)**	*pK', h*	8, 8	56.7<br>Exposed
54	S	4.248±0.434<br>(1.0)**	3.735±0.424<br>(1.0)**	*pK', h*	8, 8	80.2<br>Exposed
58	L	4.248±0.434<br>(1.0)**	3.735±0.424<br>(1.0)**	*pK', h*	7, 8	41.8<br>Part. Exposed
69	I	4.248±0.434<br>(1.0)**	3.735±0.424<br>(1.0)**	*pK', h*	7, 6	84.6<br>Exposed
70	P	4.248±0.434<br>(1.0)**	3.735±0.424<br>(1.0)**	*pK', h*	8, 6	–
72	S	4.200±0.575<br>(0.986)*	3.726±0.445<br>(0.997)**	*pK'*	8	100<br>Exposed
73	Y	3.548±1.343<br>(0.799)	3.613±0.650<br>(0.964)**	*pK'*	8	75.4<br>Exposed
74	E	4.145±0.698<br>(0.971)*	3.719±0.460<br>(0.996)**	*pK'*	8	23.8<br>NA
75	D	4.248±0.435<br>(1.0)**	3.735±0.424<br>(1.0)**	*pK'*	8	61.4<br>Exposed
77	T	4.248±0.434<br>(1.0)**	3.735±0.424<br>(1.0)**	*pK'*	8	36.6<br>NA
**Type III α neurotoxins**						
23	T	5.542±0.211<br>(1.0)**	5.519±0.166<br>(1.0)**	Esm	6	51.1<br>Exposed
26	K	5.539±0.237<br>(0.999)**	5.518±0.178<br>(1.0)**	Esm	6	49.5<br>Exposed

Site		CodeML		TreeSAAP		ASA
**27**	**G**	**5.542±0.209 (1.0)****	**5.519±0.165 (1.0)****	**Esm**	**6**	**61.9 Exposed**
**28**	**Y**	**5.542±0.209 (1.0)****	**5.519±0.165 (1.0)****	**Esm**	**6**	**88.6 Exposed**
**29**	**H**	**5.542±0.209 (1.0)****	**5.519±0.165 (1.0)****	**Esm**	**6**	**52.7 Exposed**
**30**	**D**	**5.536±0.262 (1.0)****	**5.517±0.195 (0.999)****	**Esm**	**6**	–
**35**	**K**	**5.542±0.211 (1.0)****	**5.519±0.167 (0.999)****	**Esm**	**6**	**53.4 Exposed**
36	P	5.541±0.224 (1.0)**	5.518±0.173 (1.0)**	–	–	99.7 Exposed
37	H	5.270±1.084 (0.941)	5.394±0.757 (0.973)*	–	–	75.4 Exposed
43	E	5.542±0.209 (1.0)**	5.519±0.165 (1.0)**	–	–	28.8 NA
45	F	5.542±0.209 (1.0)**	5.519±0.165 (1.0)**	–	–	22.8 NA
46	I	5.542±0.209 (1.0)**	5.519±0.165 (1.0)**	–	–	58.7 Exposed
47	P	5.540±0.232 (0.999)**	5.518±0.177 (0.999)**	–	–	96.4 Exposed
48	A	5.542±0.209 (1.0)**	5.519±0.166 (1.0)**	–	–	73.7 Exposed
49	T	5.542±0.210 (1.0)**	5.519±0.166 (1.0)**	–	–	69.1 Exposed
50	H	5.360±0.904 (0.961)*	5.438±0.620 (0.983)*	–	–	58.1 Exposed
51	G	5.214±1.172 (0.930)	5.393±0.758 (0.973)*	–	–	77.1 Exposed
52	N	5.542±0.209 (1.0)**	5.519±0.165 (1.0)**	–	–	90.3 Exposed
53	A	5.536±0.269 (0.999)**	5.516±0.201 (0.999)**	–	–	47.6 Part. Exposed
54	I	5.542±0.209 (1.0)**	5.519±0.165 (1.0)**	–	–	11.7 Buried
55	L	5.542±0.209 (1.0)**	5.519±0.165 (1.0)**	–	–	35.9 NA
56	A	5.542±0.209 (1.0)**	5.519±0.165 (1.0)**	–	–	45.2 Part. Exposed
57	R	5.196±1.205 (0.925)	5.367±0.829 (0.967)**	–	–	16 Buried
**60**	**G**	**5.542±0.211 (1.0)****	**5.519±0.166 (1.0)****	**Esm**	**6**	**22.8 NA**

Site		CodeML		TreeSAAP		ASA
**65**	**G**	**5.542±0.209 (1.0)****	**5.519±0.165 (1.0)****	**Esm**	**6**	**77.9 Exposed**
**66**	**G**	**5.542±0.209 (1.0)****	**5.519±0.166 (1.0)****	**Esm**	**6**	**100 Exposed**
**67**	**I**	**5.539±0.243 (0.999)****	**5.518±0.181 (1.0)****	**Esm**	**6**	**60.4 Exposed**
**68**	**R**	**5.542±0.209 (1.0)****	**5.519±0.165 (1.0)****	**Esm**	**6**	**12 Buried**
**69**	**P**	**5.542±0.209 (1.0)****	**5.519±0.165 (1.0)****	**Esm**	**6**	–
79	K	5.542±0.209 (1.0)**	5.519±0.165 (1.0)**	–	–	–
		**Plesiotypic α-neurotoxins from 'non-front-fanged' advanced snakes**				
21	H	3.082±0.722 (0.926)	2.484±0.206 (0.986)*	–	–	38.5 NA
22	G	3.259±0.429 (1.0)**	2.507±0.086 (1.0)**	–	–	13.4 Buried
23	F	3.246±0.460 (0.994)**	2.505±0.106 (0.998)**	–	–	46.3 Part. Exposed
25	L	3.255±0.438 (0.998)**	2.506±0.092 (0.999)**	–	–	0.4 Buried
29	–	3.039±0.773 (0.907)	2.462±0.278 (0.973)*	–	–	–
30	R	3.258±0.431 (0.999)**	2.507±0.089 (1.0)**	–	–	31.7 NA
32	T	3.161±0.619 (0.958)*	2.486±0.203 (0.987)*	–	–	58.3 Exposed
33	W	3.206±0.542 (0.977)*	2.493±0.172 (0.991)**	–	–	99 Exposed
34	S	3.258±0.431 (0.999)**	2.507±0.090 (1.0)**	–	–	52.8 Exposed
37	S	3.259±0.428 (1.0)**	2.507±0.084 (1.0)**	–	–	92.7 Exposed
38	I	3.259±0.428 (1.0)**	2.507±0.085 (1.0)**	–	–	46.6 Part. Exposed
39	G	3.257±0.433 (0.999)**	2.507±0.089 (1.0)**	–	–	9.2 Buried
40	H	3.258±0.430 (0.999)**	2.507±0.087 (1.0)**	–	–	60.6 Exposed
41	R	2.998±0.810 (0.891)	2.477±0.229 (0.982)*	–	–	82.2 Exposed
43	L	3.259±0.429 (1.0)**	2.507±0.086 (1.0)**	–	–	82.7 Exposed
44	P	3.126±0.664 (0.945)	2.492±0.175 (0.991)**	–	–	50.8 Exposed

Site		CodeML		TreeSAAP		ASA
46	H	3.032±0.776 (0.906)	2.482±0.214 (0.984)*	–	–	26.2 NA
47	M	3.259±0.429 (1.0)**	2.507±0.086 (1.0)**	–	–	52.7 Exposed
48	T	3.187±0.574 (0.970)*	2.496±0.158 (0.993)**	–	–	22.5 NA
53	Y	3.149±0.634 (0.954)*	2.491±0.179 (0.990)**	–	–	31.6 NA
54	K	3.190±0.568 (0.971)*	2.499±0.142 (0.995)**	–	–	41.3 Part. Exposed
55	P	**3.258±0.432 (0.999)****	**2.507±0.088 (1.0)****	**V^o**	**6**	40.9 **Part. Exposed**
56	D	**3.197±0.556 (0.974)***	**2.500±0.136 (0.995)****	**V^o**	**6**	46.2 **Part. Exposed**
57	E	**2.879±0.907 (0.843)**	**2.473±0.241** (0.979)*	**V^o**	**6**	88.3 **Exposed**
58	N	**3.215±0.524 (0.981)***	**2.499±0.140** (0.995)**	**V^o**	**6**	86.5 **Exposed**
64	A	**3.241±0.471 (0.992)****	**2.504±0.110** (0.998)**	**V^o**	**6**	32.4 NA
70	R	3.259±0.429 (1.0)**	2.507±0.086 (1.0)**	–	–	52.2 Exposed
71	M	3.073±0.732 (0.922)	2.485±0.203 (0.986)*	–	–	90.6 Exposed
74	T	**3.217±0.521 (0.982)***	**2.501±0.130** (0.996)**	**V^o**	**6**	100 **Exposed**
76	K	**3.258±0.432 (0.999)****	**2.507±0.089 (1.0)****	**V^o**	**6**	82 **Exposed**
77	S	**3.084±0.720 (0.927)**	**2.483±0.210** (0.985)*	**V^o**	**6**	100 **Exposed**
80	R	**2.457±1.092 (0.670)**	**2.426±0.353** (0.951)*	**V^o**	**6**	64.8 **Exposed**
85	T	**3.256±0.437 (0.998)****	**2.506±0.092** (0.999)**	**V^o**	**6**	83.4 **Exposed**
86	G	**3.257±0.434 (0.999)****	**2.506±0.091 (1.0)****	**V^o**	**6**	49.7 **Exposed**
88	S	3.131±0.658 (0.947)	2.493±0.171 (0.991)**	–	–	34.8 NA
91	S	3.259±0.428 (1.0)**	2.507±0.085 (1.0)**	–	–	64.7 Exposed
92	D	2.905±0.896 (0.850)	2.432±0.351 (0.954)**	–	–	34.9 NA
**Plesiotypic α-neurotoxins from Elapidae**						
7	S	3.499±0.841 (0.919)	3.474±0.525 (0.969)*	–	–	–

Site		CodeML		TreeSAAP		ASA
28	S	3.485±0.864<br>(0.913)	3.451±0.581<br>(0.961)*	–	–	77.6<br>Exposed
30	Y	3.692±0.532<br>(0.986)*	3.549±0.296<br>(0.996)**	–	–	63.0<br>Exposed
33	P	3.731±0.433<br>(0.999)**	3.56±0.245<br>(1.0)**	–	–	66.5<br>Exposed
34	N	3.694±0.529<br>(0.986)*	3.549±0.297<br>(0.996)**	–	–	87.7<br>Exposed
35	S	3.597±0.707<br>(0.953)*	3.518±0.407<br>(0.985)*	–	–	85.8<br>Exposed
39	P	3.69±0.54<br>(0.985)*	3.545±0.314<br>(0.994)**	–	–	85.3<br>Exposed
40	D	3.724±0.455<br>(0.996)**	3.558±0.255<br>(0.999)**	–	–	66.1<br>Exposed
44	I	3.718±0.47<br>(0.994)**	3.556±0.264<br>(0.998)**	–	–	14.8<br>Buried
48	R	3.68±0.561<br>(0.981)*	3.543±0.32<br>(0.994)**	–	–	57.5<br>Exposed
50	W	3.549±0.776<br>(0.937)	3.498±0.461<br>(0.978)*	–	–	41.3<br>Par. exposed
52	T	3.729±0.44<br>(0.998)**	3.559±0.248<br>(1.0)**	–	–	65.8<br>Exposed
53	A	3.724±0.453<br>(0.997)**	3.558±0.254<br>(0.999)**	–	–	91.8<br>Exposed
54	V	3.733±0.426<br>(1.0)**	3.56±0.242<br>(1.0)**	–	–	96.8<br>Exposed
55	R	3.712±0.486<br>(0.992)**	3.553±0.276<br>(0.998)**	–	–	100.0<br>Exposed
57	R	3.721±0.463<br>(0.995)**	3.557±0.259<br>(0.999)**	–	–	42.2<br>Par. exposed
58	E	3.695±0.526<br>(0.986)*	3.549±0.294<br>(0.996)**	–	–	87.0<br>Exposed
59	I	3.399±0.948<br>(0.885)	3.43±0.616<br>(0.954)*	–	–	9.3<br>Buried
60	R	3.733±0.427<br>(1.0)**	3.56±0.242<br>(1.0)**	–	–	91.7<br>Exposed
69	P	3.708±0.495<br>(0.991)**	3.553±0.277<br>(0.997)**	–	–	91.7<br>Exposed
70	S	3.715±0.477<br>(0.993)**	3.555±0.27<br>(0.998)**	–	–	11.8<br>Buried
72	L	3.712±0.486<br>(0.992)**	3.554±0.275<br>(0.998)**	–	–	88.4<br>Exposed
73	G	3.65±0.622<br>(0.971)*	3.533±0.358<br>(0.990)**	–	–	54.0<br>Exposed

Site		CodeML		TreeSAAP		ASA
74	L	3.687±0.543 (0.984)*	3.546±0.306 (0.995)**	–	–	25.2 NA
75	T	3.722±0.459 (0.996)**	3.557±0.258 (0.999)**	–	–	39.8 Par. exposed
77	F	3.725±0.451 (0.997)**	3.558±0.254 (0.999)**	–	–	42.7 Par. exposed
83	N	3.602±0.697 (0.955)*	3.523±0.389 (0.987)*	–	–	69.0 Exposed
86	H	3.733±0.428 (1.0)**	3.56±0.243 (1.0)**	–	–	100.0 Exposed
**Viperidae 3FTxs**						
7	I	6.037±0.994 (0.983)*	5.932±0.85 (0.991)**	–	–	–
**18**	**S**	**5.874±1.314 (0.953)***	**5.825±1.11 (0.971)***	***El*, α*c***	**7, 6**	–
**23**	**E**	**5.796±1.430 (0.939)**	**5.784±1.186 (0.963)***	***El*, α*c***	**7, 6**	–
**25**	**Y**	**6.123±0.76 (0.999)****	**5.979±0.705 (1.0)****	***El*, α*c***	**7, 6**	–
**28**	**N**	**5.995±1.086 (0.975)***	**5.908±0.914 (0.986)***	***El, Pr***	**7, 8**	–
**29**	**M**	**6.128±0.744 (1.0)****	**5.981±0.697 (1.0)****	***El, Pr***	**7, 8**	–
**30**	**T**	**6.128±0.745 (1.0)****	**5.981±0.698 (1.0)****	***El, Pr***	**7, 8**	–
**31**	**F**	**6.12±0.77 (0.998)****	**5.977±0.71 (0.999)****	***El, Pr***	**6, 8**	–
**34**	**L**	**6.128±0.745 (1.0)****	**5.981±0.698 (1.0)****	***El, Pr***	**6, 8**	–
**36**	**R**	**6.122±0.766 (0.999)****	**5.978±0.709 (0.999)****	***El, Pr***	**6, 8**	–
**40**	**E**	**6.004±1.065 (0.977)***	**5.913±0.9 (0.987)***	***El, Pr***	**8, 7**	**100 Exposed**
**42**	**L**	**6.07±0.913 (0.989)***	**5.952±0.792 (0.995)****	***El, Pr***	**8**	**32.0 NA**
**49**	**K**	**6.042±0.984 (0.984)***	**5.934±0.844 (0.991)****	***El***	**8**	**48.8 Par. Exposed**
**51**	–	**6.126±0.753 (0.999)****	**5.98±0.702 (1.0)****	***El***	**8**	**100 Exposed**
54	L	5.757±1.510 (0.932)	5.716±1.325 (0.951)*	–	–	–
55	F	6.106±0.815 (0.996)**	5.969±0.738 (0.998)**	–	–	16.1 Buried
56	P	6.037±0.995 (0.983)*	5.932±0.851 (0.991)**	–	–	100 Exposed

Site		CodeML		TreeSAAP		ASA
57	V	5.757±1.485 (0.932)	5.754±1.241 (0.958)*	–	–	91.3 Exposed
58	L	5.965±1.156 (0.969)*	5.878±0.996 (0.980)*	–	–	42.9 Par. Exposed
59	K	5.818±1.400 (0.943)	5.795±1.166 (0.965)*	–	–	64.2 Exposed
61	E	5.780±1.449 (0.936)	5.775±1.199 (0.962)*	–	–	55.2 Exposed
**70**	**Q**	**5.939±1.205 (0.965)***	**5.863±1.029 (0.978)***	***Pr***	7	**100.0 Exposed**
**72**	**W**	**6.127±0.75 (1.0)****	**5.98±0.7 (1.0)****	***Pr*, α*c***	7, 6	**98.5 Exposed**
**73**	**T**	**6.119±0.775 (0.998)****	**5.977±0.712 (0.999)****	***Pr*, α*c***	7 , 6	**43.5 Par. Exposed**
**74**	**D**	**6.069±0.913 (0.989)***	**5.953±0.788 (0.995)****	***Pr*, α*c***	7 , 6	**2.8 Buried**
**75**	**K**	**5.802±1.430 (0.940)**	**5.773±1.212 (0.961)***	***El*, *Pr*, α*c***	**8, 7 , 6**	**92.4 Exposed**
**78**	**E**	**6.109±0.804 (0.996)****	**5.972±0.728 (0.998)****	***El*, *Pr*, α*c***	**8, 7 , 6**	**70.8 Exposed**
**80**	**N**	**5.885±1.287 (0.955)***	**5.848±1.053 0.975***	***El*, α*c***	**8, 6**	**98.7 Exposed**
**81**	**K**	**6.112±0.795 (0.997)****	**5.973±0.724 (0.999)****	***El*, α*c***	**8, 6**	**83.2 Exposed**
84	I	6.127±0.747 (1.0)**	5.981±0.698 (1.0)**	–	–	–
			**κ-bungarotoxins**			
37	Q	8.983±1.705 0.982*	8.708±1.708 (0.989)*	–	–	49.1 Par. Exposed
44	L	9.069±1.500 0.993**	8.761±1.593 (0.995)**	–	–	40 Par. Exposed
47	Q	9.121±1.355 (0.999)**	8.792±1.515 (0.999)**	–	–	57.6 Exposed
50	K	8.956±1.761 0.979*	8.692±1.74 (0.987)*	–	–	39.8 Par. Exposed
53	S	9.126±1.341 (1.0)**	8.795±1.507 (1.0)**	–	–	62.7 Exposed
			**Cytotoxins**			
50	T	7.473±1.990 (0.996)**	6.602±2.042 (0.999)**	*P*α, K^0	6, 7	75.2 Exposed
51	P	7.435±2.057 (0.988)*	6.590±2.062 (0.996)**	*P*α, K^0	6, 7	45.7 Part. exposed

Amino-acid property symbols used: α–helical tendencies (*P*α), Compressibility (K^0),Equalibrium constant (ionization of COOH) *(pK')*, Hydropathy *(h)*, Long-range n.b. energy *(El)*, Normalized consensus hydrophobicity *(Hnc)*, Partial specific volume *(V^0)*, Polar requirement *(Pr)*, Power to be at C-terminus of α-helix (αc), Short and medium-range n.b. energy *(Esm)*, Solvent accessible reduction ratio *(R*α*)* and Surrounding hydrophobicity *(Hp)*.

Legend:

a: M2a Bayes Empirical Bayes (BEB) posterior probability (* ≥ 0.95; ** ≥ 0.99) and post-mean omega indicated in brackets

b: M8 Bayes Empirical Bayes (BEB) posterior probability (* ≥ 0.95; ** ≥ 0.99) and post-mean omega indicated in brackets

c: amino acid property under selection

d: magnitude of selection on the amino acid property

ASA: Accessible surface area (50% ≥ Side chains completely exposed; 20% ≤ Side chains buried)

Part. exposed: Partially exposed side-chains (ASA: 40%-50%)

Sites detected as positively selected by both nucleotide and amino acid-level analyses are indicated in bold.

Supplementary Table 12. Surface accessibility of Three-finger toxins

Type I α-neurotoxin

**Total Residues**	62
**Total Exposed**	33
**Total Buried**	10
**Exposed PS**	15 (24%)
**Buried PS**	2 (3%)
**Frequency of exposed PS sites (a)**	45%
**Frequency of buried PS sites (b)**	20%
**Exposure Ratio (a/b)**	**2.3%**

Type II α-neurotoxin

**Total Residues**	74
**Total Exposed**	29
**Total Buried**	20
**Exposed PS**	14 (19%)
**Buried PS**	3 (4%)
**Frequency of exposed PS sites (a)**	48%
**Frequency of buried PS sites (b)**	15%
**Exposure Ratio (a/b)**	**3.2%**

Type III α-neurotoxin

**Total Residues**	57
**Total Exposed**	28
**Total Buried**	14
**Exposed PS**	20 (35%)
**Buried PS**	3 (5%)
**Frequency of exposed PS sites (a)**	71%
**Frequency of buried PS sites (b)**	21%
**Exposure Ratio (a/b)**	**3.3%**

'Non-front-fanged' Advanced Snakes

**Total Residues**	74
**Total Exposed**	32
**Total Buried**	17
**Exposed PS**	25 (34%)
**Buried PS**	3 (4%)
**Frequency of exposed PS sites (a)**	78%
**Frequency of buried PS sites (b)**	18%
**Exposure Ratio (a/b)**	**4.4%**

Viperidae

**Total Residues**	46
**Total Exposed**	23
**Total Buried**	8
**Exposed PS**	15 (33%)
**Buried PS**	2 (4%)
**Frequency of exposed PS sites (a)**	65%
**Frequency of buried PS sites (b)**	25%
**Exposure Ratio (a/b)**	**2.6%**

Elapidae

**Total Residues**	65
**Total Exposed**	32
**Total Buried**	14
**Exposed PS**	23 (35%)
**Buried PS**	3 (5%)
**Frequency of exposed PS sites (a)**	72%
**Frequency of buried PS sites (b)**	21%
**Exposure Ratio (a/b)**	**3.4%**

κ-neurotoxins

**Total Residues**	132
**Total Exposed**	47
**Total Buried**	42
**Exposed PS**	3 (2%)
**Buried PS**	0
**Frequency of exposed PS sites (a)**	6%
**Frequency of buried PS sites (b)**	0
**Exposure Ratio (a/b)**	–

Cytotoxins

**Total Residues**	60
**Total Exposed**	28
**Total Buried**	15
**Exposed PS**	2 (3%)
**Buried PS**	0
**Frequency of exposed PS sites (a)**	7%
**Frequency of buried PS sites (b)**	0
**Exposure Ratio (a/b)**	–

Legend: PS: Positively selected

Note:

- Type I α-neurotoxins had 39.4% of the exposed residues and 20% of the buried residues under positive selection. Thus, the exposed residues being **1.9 times** more likely to be positively selected than buried residues.
- Type II α-neurotoxins had 36.8% of the exposed residues and 15% of the buried residues under positive selection. Thus, the exposed residues being **2.5 times** more likely to be positively selected than buried residues.
- Type III α-neurotoxin had 58.8% of the exposed residues and 21.4% of the buried residues under positive selection. Thus, the exposed residues being **2.7 times** more likely to be positively selected than buried residues.
- The plesiotypic 'non-front-fanged' advanced snake 3FTx had 62.5% of the exposed residues and 17.6% of the buried residues under positive selection. Thus, the exposed residues being **3.5 times** more likely to be positively selected than buried residues.
- The plesiotypic Viperidae 3FTx had 51.7% of the exposed residues and 25% of the buried residues under positive selection. Thus, the exposed residues being **2.1 times** more likely to be positively selected than buried residues.
- The plesiotypic Elapidae 3FTx had 58.9% of the exposed residues positively selected while 21.4% of the buried residues under positive selection. Thus, the exposed residues being **2.8 times** more likely to be positively selected than buried residues.
- κ-neurotoxins had 5% of the exposed residues under positive selection, while none of the buried residues were positively selected.
- Cytotoxic 3FTx had 5.8% of the exposed residues under positive selection, while none of the buried residues were positively selected.

Supplementary Table 13. Structural and functional residues in 3FTx

3FTx	Structurally and/or functionally important residue	Reference
**α-neurotoxins**		
**Type I (short-chain)**	Q6 (87%), S8 (95%), S9 (95%), Q10 (87%), Y25 (invariant), K27 (94%), W29 (95%), D31 (91%), R33 (89%), G34 (94%), E38 (99%), G40 (invariant), P44 (99%), K47 (93%)	(Pillet et al. 1993; Tremeau et al. 1995; Antil et al. 1999; Barber et al. 2013)
**Type II (long-chain)**	Y21 (72%), K23 (84%), W25 (98%), C26 (94%), D27 (91%), A28 (50%; PS), F29 (49%; PS), C30 (94%), R33 (83%), K35 (73%), R36 (very low; PS), G40 (invariant), P46 (98%), K49 (70%), F65 (very low; PS)	(Antil et al. 1999; Antil-Delbeke et al. 2000; Barber et al. 2013)
**Type III**	L1 (invariant), T20 (98%), S43 (90%), V51 (91%), S54 (91%), T55 (93%), D56 (invariant), N59 (invariant)	Predicted in this study as putative functional/structural residue
**Plesiotypic 'non-front-fanged' advanced snakes**	E64 (95%), N75 (95%)	Predicted in this study as putative functional/structural residue
**Plesiotypic Elapidae 3FTx**	K28 (invariant), R42 (invariant), P49 (invariant), V57 (96%), T62 (93%), D63 (invariant), N66 (96%)	Predicted in this study as putative functional/structural residue
**Viperidae 3FTx**	P20 (90%), G45 (invariant), K59 (90%), T66 (90%), N70 (invariant)	Predicted in this study as putative functional/structural residue
**κ-bungarotoxin**	R32 (invariant), P45 (invariant), F47 (invariant), L55 (invariant)	(Dewan et al. 1994)
**Cytotoxin**	K5 (83%), Y11 (72%), K12 (invariant), K18 (97%), Y22 (invariant), K23 (invariant), M24 (94%), M26 (invariant), K31 (56%), K35 (invariant), G37 (invariant), P43 (invariant), K44 (invariant), K50 (invariant), Y51 (invariant)	(Kumar et al. 1997)

PS: Positively selected.

Percent identity: has been indicated in parenthesis for each site.

Very low: very few sequences have the structurally/functionally important residue at this site.

Note: All site numbering corresponds to H8PG58 for short-chain, FJ752458.1for long-chain, AF082975.1 for Type III, DQ366293.1 for basal 3FTx from 'non-front-fanged' advanced snakes, AY611643.1 for basal 3FTx from Elapidae, AY057872.1 for kappa and U42585.1 for cytotoxins.

References

Antil S, Servent D, Menez A. 1999. Variability among the sites by which curaremimetic toxins bind to torpedo acetylcholine receptor, as revealed by identification of the functional residues of alpha-cobratoxin. J Biol Chem 274:34851–34858.

Antil-Delbeke S, Gaillard C, Tamiya T, Corringer PJ, Changeux JP, Servent D, Ménez a. 2000. Molecular determinants by which a long chain toxin from snake venom interacts with the neuronal alpha 7-nicotinic acetylcholine receptor. The Journal of biological chemistry 275:29594–29601.

Barber CM, Isbister GK, Hodgson WC. 2013. Alpha neurotoxins. Toxicon : official journal of the International Society on Toxinology 66C:47–58.

Dewan JC, Grant GA, Sacchettini JC. 1994. Crystal structure of kappa-bungarotoxin at 2.3-A resolution. Biochemistry 33:13147–13154.

Kumar TK, Jayaraman G, Lee CS, Arunkumar AI, Sivaraman T, Samuel D, Yu C. 1997. Snake venom cardiotoxins-structure, dynamics, function and folding. Journal of biomolecular structure dynamics 15:431–463.

Pillet L, Tremeau O, Ducancel F, Drevet P, Zinn-Justin S, Pinkasfeld S, Boulain JC, Menez A. 1993. Genetic engineering of snake toxins. Role of invariant residues in the structural and functional properties of a curaremimetic toxin, as probed by site-directed mutagenesis. J Biol Chem 268:909–916.

Tremeau O, Lemaire C, Drevet P, Pinkasfeld S, Ducancel F, Boulain JC, Menez A. 1995. Genetic engineering of snake toxins. The functional site of Erabutoxin a, as delineated by site-directed mutagenesis, includes variant residues. J Biol Chem 270:9362–9369.
